# DRD1 downregulation contributes to mechanical stretch-induced lung endothelial barrier dysfunction

**DOI:** 10.7150/thno.46192

**Published:** 2021-01-01

**Authors:** Yan Wang, Yu-Jian Liu, Dun-Feng Xu, Hui Zhang, Chu-Fan Xu, Yan-Fei Mao, Zhou Lv, Xiao-Yan Zhu, Lai Jiang

**Affiliations:** 1 Department of Anesthesiology and Surgical Intensive Care Unit, Xinhua Hospital, Shanghai Jiaotong University School of Medicine, Shanghai, 200092, China.; 2 School of Kinesiology, The key Laboratory of Exercise and Health Sciences of Ministry of Education, Shanghai University of Sport, Shanghai, 200438, China.; 3 Department of Physiology, Navy Medical University, Shanghai, 200433, China.

**Keywords:** DRD1, mechanical ventilation, cyclic stretch, microtubule, pulmonary vascular endothelial cell

## Abstract

**Rationale:** The lung-protective effects of dopamine and its role in the pathology of ventilator-induced lung injury (VILI) are emerging. However, the underlying mechanisms are still largely unknown.

**Objective:** To investigate the contribution of dopamine receptor dysregulation in the pathogenesis of VILI and therapeutic potential of dopamine D1 receptor (DRD1) agonist in VILI.

**Methods:** The role of dopamine receptors in mechanical stretch-induced endothelial barrier dysfunction and lung injury was studied in DRD1 knockout mice, in isolated mouse lung vascular endothelial cells (MLVECs), and in lung samples from patients who underwent pulmonary lobectomy with mechanical ventilation for different time periods.

**Measurements and Main Results:** DRD1 was downregulated in both surgical patients and mice exposed to mechanical ventilation. Prophylactic administration of dopamine or DRD1 agonist attenuated mechanical stretch-induced lung endothelial barrier dysfunction and lung injury. By contrast, pulmonary knockdown or global knockout of DRD1 exacerbated these effects. Prophylactic administration of dopamine attenuated mechanical stretch-induced α-tubulin deacetylation and subsequent endothelial hyperpermeability through DRD1 signaling. We identified that cyclic stretch-induced glycogen-synthase-kinase-3β activation led to phosphorylation and activation of histone deacetylase 6 (HDAC6), which resulted in deacetylation of α-tubulin. Upon activation, DRD1 signaling attenuated mechanical stretch-induced α-tubulin deacetylation and subsequent lung endothelial barrier dysfunction through cAMP/exchange protein activated by cAMP (EPAC)-mediated inactivation of HDAC6.

**Conclusions:** This work identifies a novel protective role for DRD1 against mechanical stretch-induced lung endothelial barrier dysfunction and lung injury. Further study of the mechanisms involving DRD1 in the regulation of microtubule stability and interference with DRD1/cAMP/EPAC/HDAC6 signaling may provide insight into therapeutic approaches for VILI.

## Introduction

Mechanical ventilation is a critical intervention for patients with acute respiratory failure 1, 2. However, lung overdistension induced by mechanical ventilation also causes pulmonary endothelial dysfunction 3. The injurious effect of mechanical stretch on pulmonary endothelium has been implicated in the development of ventilator-induced lung injury (VILI), which is characterized by pulmonary inflammation and particularly increased vascular permeability 3, 4. In addition, we and others have previously shown that mechanical stretch increases cultured lung endothelial monolayer permeability *in vitro* and promotes lung vascular permeability in mice 5-7. Thus, elucidating the mechanisms underlying the mechanical stretch-induced lung endothelial barrier dysfunction may provide insight into novel clinical therapeutics for VILI.

Microtubules are rope-like polymers of α- and β-tubulin. As a key component of the cytoskeleton, microtubules play a critical role in maintaining cell morphology and endothelial barrier integrity 8-10. Destabilization of the microtubule network has been shown to result in actin stress fiber formation and contraction in pulmonary endothelial cells, leading to endothelial barrier disruption and increased permeability 9-11. Microtubules cycle between polymerized and depolymerized states and this dynamic is controlled by diverse post-translational modifications such as acetylation and detyrosination, which may reflect stability of the microtubule network 12, 13. Indeed, acetylated α-tubulin has been associated with stable microtubule 14. The acetylation level of α-tubulin is largely modulated by tubulin deacetylases such as histone deacetylase 6 (HDAC6) and sirtuin-2 (SIRT2), both of which deacetylate the Lysine-40 residue of α-tubulin, *in vitro* and *in vivo* 15, 16. The inhibition of either HDAC6 or SIRT2 by siRNA or specific inhibitors induces hyperacetylation of the microtubule network. Previous studies have identified that microtubule disassembly contributes to endothelial barrier leakage during VILI 11, 17. However, the mechanisms involved in the mechanical stretch-induced deregulation of microtubule stability remain poorly understood.

Dopamine is a neurotransmitter, that can be produced outside the central nervous system. Lung alveolar epithelial cells represent an important source of extraneural dopamine, which has a significant role in local organ physiology 18. In addition, a recent study has suggested sympathetic innervations as a neural source of dopamine in the lung 19. Dopamine D1 receptor (DRD1) and D2 receptor (DRD2) are present in lung tissues. Activation of DRD2 induces Na-K-ATPase gene expression 20. Moreover, activation of DRD1 results in the trafficking of Na-K-ATPase to the basolateral membrane of type II alveolar epithelial cells, thus increasing lung liquid clearance during acute lung injury (ALI) 21, 22. Although the lung-protective effects of dopamine and its role in the pathology of ALI are emerging, the mechanisms are still largely unknown.

In the present study, we demonstrate that dopamine acts as an endogenous regulator of microtubule stability through modulating HDAC6-regulated α-tubulin acetylation in lung microvascular endothelial cells. Upon activation, DRD1 signaling attenuates mechanical stretch-induced α-tubulin deacetylation and subsequent lung endothelial barrier dysfunction via inactivation of HDAC6. Our findings suggest DRD1 as a potential target for the treatment of mechanical stretch-induced lung endothelial barrier dysfunction.

## Methods

### Lung Tissues of Patients with Intraoperative Mechanical Ventilation Support

The study was approved by the Ethics Committee of Xinhua Hospital, Shanghai Jiaotong University of Medicine, and informed consent was obtained from all patients. This research project is a clinical trial registered in Clinicaltrials.gov (NCT03317431; https://clinicaltrials.gov/ct2/show/NCT03317431). Adult patients who were to undergo elective pulmonary lobectomy with general anesthesia and mechanical ventilation and classified as physical status I to III according to the American Society of Anesthesiologists Physical Status Classification System were eligible for this study. Exclusion criteria included a history of any lung disease or distant metastases, recent anesthetics or mechanical ventilation treatment, and participation in another clinical trial. All patients were general anesthetized with double endotracheal tube intubation and received the same mechanical ventilation protocol, consisting of 6-8 mL/kg tidal volume, 5 cm H_2_O positive end-expiratory pressure, 40% inspired oxygen concentration, 10-15/min respiratory rate, and an inspiratory to expiratory ratio of 1:2. Ventilation protocol was allowed to change at any time point if there was any concern about patient's safety or upon the surgeon's request, but patients with changed ventilation protocol were removed from this study. The investigators recorded the time period from the beginning of mechanical ventilation to one-lung ventilation. After lung resection surgery, non-tumor tissues from the excised lung specimen were cut into small pieces, washed with saline to remove any blood, transferred to a clean tube, immediately frozen in liquid nitrogen, and stored at -80 °C until use.

Detailed description of further methods is provided in the online supplement.

## Results

### Mechanical ventilation leads to a decrease in pulmonary DRD1 expression in surgical patients, which is negatively correlated with the duration of ventilation

Dopamine is synthesized from tyrosine through the following two steps: Tyrosine is first converted to 3, 4-dihydroxy-l-phenylalanine (L-DOPA) by tyrosine hydroxylase (TH), and L-DOPA is then converted to dopamine by L-DOPA decarboxylase (DDC) 23. We first observed the localization of DRD1, DRD2 and dopamine synthetic enzymes in non-nervous cells including endothelial cells, epithelial cells and fibroblasts in lung tissues. As shown in Figure S1, double immunofluorescence staining in human and mouse lung tissue sections revealed that DRD1, DRD2, as well as dopamine synthetic enzymes TH and DDC were co-localized with vascular endothelial cell marker CD31, epithelial cell marker E-cadherin and fibroblast marker PDGFRα. In addition, we sorted whole lungs of mice based on cell-surface marker presentation (Figure S2A-D) and examined mRNA levels of DRD1, DRD2, TH and DDC in populations of endothelial cells (CD31^+^, PDGFRα^-^, EPCAM^-^ and CD45^-^), epithelial cells (EPCAM^+^, PDGFRα^-^, CD31^-^ and CD45^-^) and fibroblasts (PDGFRα^+^, EPCAM^-^, CD31^-^ and CD45^-^). The results shown in Figure S2E-H further confirmed the expression of DRD1, DRD2, TH and DDC in endothelial cells, epithelial cells and fibroblasts in healthy adult lungs.

**Figure 1:**
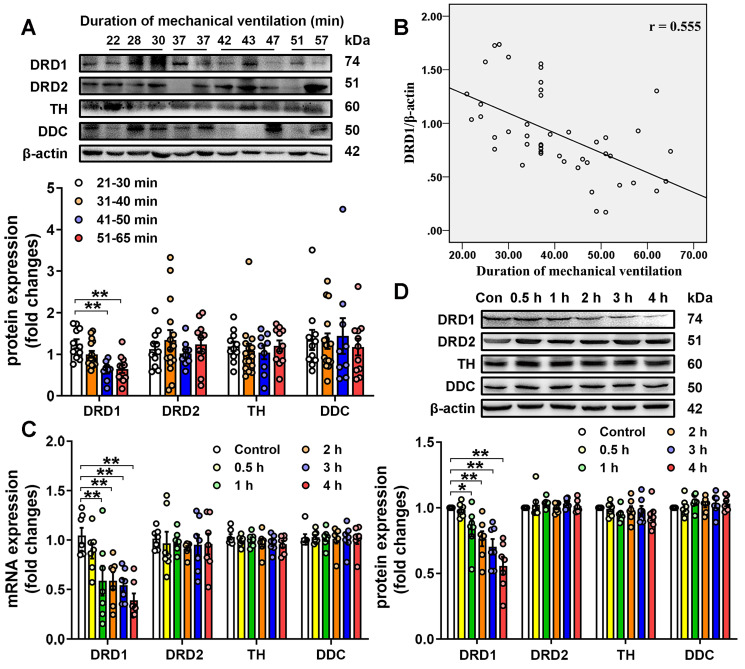
** Expression of dopamine receptors and dopamine synthetic enzymes in the lungs. A&B**, Lung tissue were harvested from ventilated patients (n = 11 in 21-30 min group, n = 15 in 31-40 min group, n = 9 in 41-50 min group, n = 11 in 51-65 min group). **(A)** Protein expression of DRD1, DRD2, TH and DDC in lung homogenates was determined by Western blot analysis and the representative protein bands are presented on the top of corresponding histograms. **(B)** The correlation between duration of mechanical ventilation and DRD1 expression. **C&D**, Mice were subjected to mechanical ventilation (30 mL/kg) and lung tissues were harvested at the time points indicated (n = 7). Quantitative real-time reverse-transcription polymerase chain reaction and Western blot analysis were used to determine mRNA and protein expression of DRD1, DRD2, TH and DDC. Representative protein bands were presented on the top of corresponding histograms. Data are expressed as means ± SEM. * p < 0.05, ** p < 0.01.

We then investigated the effect of mechanical ventilation on the expression of DRD1, DRD2, TH and DDC in lung tissues harvested from surgical patients with intraoperative mechanical ventilation support. A total of 46 surgical patients were recruited during the study period. Patients were divided into four groups according to the duration of mechanical ventilation, and the demographics were shown in Table S1. As shown in Figure 1A, patients who received longer ventilation (mechanical ventilation > 41 min) had significantly lower DRD1 levels compared with those who received shorter ventilation (mechanical ventilation < 40 min). However, there were no significant differences in the pulmonary levels of DRD2, TH and DDC among the four groups of patients. To further assess the impact of mechanical ventilation, the protein levels of DRD1, DRD2, TH and DDC were plotted according to the duration of mechanical ventilation. As shown in Figure 1B, we found that pulmonary levels of DRD1, but not DRD2, TH or DDC, were negatively correlated with the duration of mechanical ventilation.

### Prophylactic treatment of dopamine alleviates mechanical ventilation-induced lung vascular hyperpermeability and acute lung injury via DRD1 signaling

We then examined the effect of mechanical stretch on the pulmonary levels of DRD1, DRD2, TH and DDC in a murine model of mechanical ventilation-induced lung injury. As shown in Figure 1C-D, 1-h mechanical ventilation resulted in a significant, time-dependent decrease in mRNA and protein levels of DRD1, but not DRD2, TH and DDC in lung tissues. In addition, mechanical ventilation did not result in changes in the protein levels of DRD1 and DRD2 in extra-pulmonary tissues, including heart, brain and kidney (Figure S3). To clarify why mechanical ventilation attenuated DRD1, but not DRD2, we focused on microRNAs (miRs), which negatively regulated the target mRNAs 24. We found that potential DRD1-targeting miRs (miR-30c-5p, miR-181c-5p and miR-302a-3p) were different from potential DRD2-targeting miRs (Table S2). As shown in Figure S4, mechanical ventilation increased pulmonary expression of miR-302a-3p, but not miR-30c-5p, miR-181c-5p. Furthermore, miR302a-3p mimic dose-dependently decreased DRD1 mRNA and protein expression, whereas had no significant effect on DRD2 expression in primary cultured mouse lung vascular endothelial cells (MLVECs). These results suggest mechanical ventilation may selectively downregulate DRD1 through a miR-302a-3p-dependent mechanism.

The protective effects of dopamine against ventilation-induced acute lung injury have been reported in previous studies 25, 26. To examine whether downregulation of DRD1 expression contributed to mechanical ventilation-induced lung injury, we applied an siRNA approach targeting DRD1 and DRD2 and observed its effect on dopamine protection against VILI. Upon intratracheal transfection with siRNAs targeting DRD1 and DRD2, the pulmonary levels of DRD1 and DRD2 decreased by approximately 73% and 74%, respectively, whereas DRD1 and DRD2 levels in extra-pulmonary tissues, including heart, brain and kidney, remained unchanged (Figure S5). To further clarify whether endothelial DRD1 and DRD2 expression was really suppressed by intratracheally administered siRNA, we sorted whole lungs of mice isolated 72 h after siRNA administration based on cell-surface marker presentation (Figure S2A-D) and examined mRNA levels of DRD1 and DRD2 in populations of epithelial and endothelial cells. As shown in Figure S6, intratracheal transfection with DRD1 siRNA resulted in approximately 83% and 67% decreases in DRD1 mRNA levels in pulmonary epithelial and endothelial cells, respectively. Intratracheal transfection with DRD2 siRNA led to about 87% and 71% decreases in DRD2 mRNA levels in pulmonary epithelial and endothelial cells, respectively.

**Figure 2:**
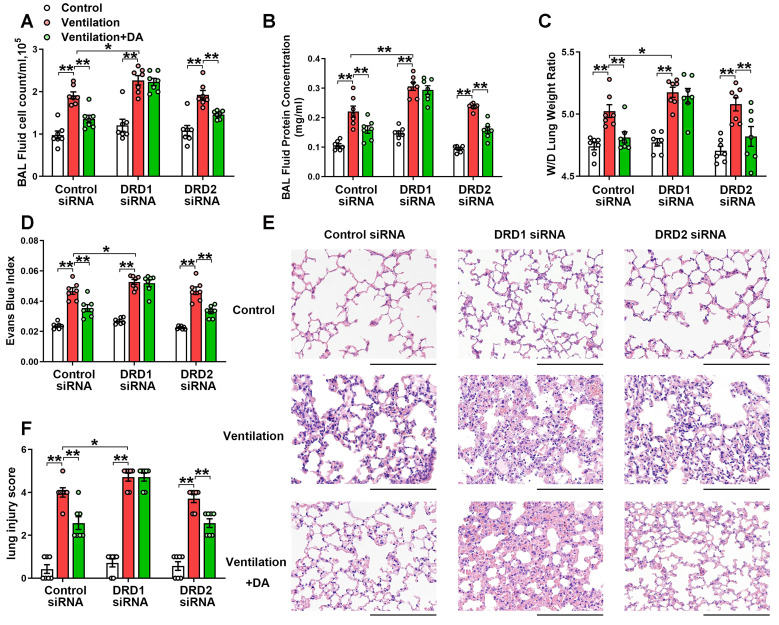
** DRD1 siRNA, but not DRD2 siRNA, blocks the protective effect of dopamine against mechanical ventilation-induced acute lung injury.** Mice were intratracheally instilled with control or DRD1/DRD2 siRNA (2 mg/kg). Seventy-two hours later, mice were subjected to mechanical ventilation (30 mL/kg) for 4 h. Dopamine (50 mg/kg) was intraperitoneally administered before the onset of ventilation. **(A)** Cell count and **(B)** protein concentration were performed in BAL fluid. **(C)** Lung W/D ratio was measured as an index of pulmonary edema. **(D)** Pulmonary vascular permeability was analyzed by using Evans blue-labeled albumin extravasation into the lung tissue. **(E)** The left lower lung was used for histological evaluation by H&E staining. Original magnification, × 200. Scale bar= 100 μm. **(F)** The severity of lung injury was scored to quantify the severity of lung pathology. Data are expressed as means ± SEM (n = 7). * p < 0.05, ** p < 0.01.

To observe the effect of dopamine on VILI, dopamine (50 mg/kg) was intraperitoneally administered before the onset of mechanical ventilation. As expected, prophylactic treatment of dopamine significantly alleviated mechanical ventilation-induced increases in bronchoalveolar lavage (BAL) cell counts and protein levels (Figure 2A-B). Mechanical ventilation led to increased lung wet/dry weight ratio (W/D) and Evans blue dye leakage from the vascular space into the lung parenchyma, which was significantly alleviated by dopamine treatment (Figure 2C-D). Histopathological analysis of H&E-stained lung tissue sections showed that prophylactic treatment of dopamine significantly mitigated mechanical ventilation-induced diffuse interstitial edema, alveolar thickening, alveolar air space narrowing and lung recruitment of leukocytes, with a reduced lung injury score (Figure 2E-F). These findings strongly indicate that prophylactic treatment of dopamine attenuates VILI-associated lung vascular hyperpermeability *in vivo*.

**Figure 3:**
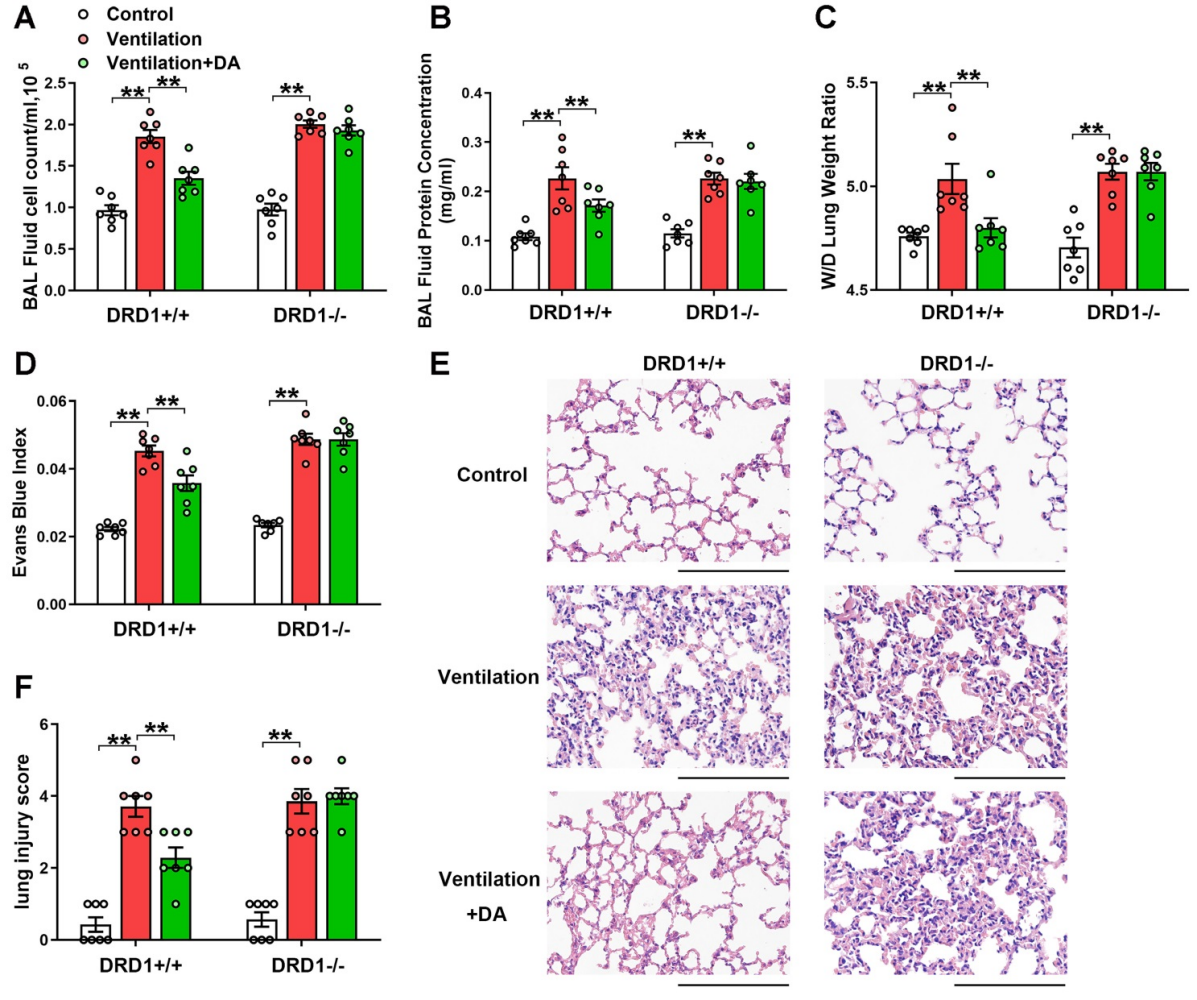
** DRD1 deficiency abrogates the protective effect of dopamine against mechanical ventilation-induced acute lung injury.** Wide-type (DRD1^+/+^) and DRD1 knockout (DRD1^-/-^) mice were subjected to mechanical ventilation (30 mL/kg) for 4 h. Dopamine (50 mg/kg) was intraperitoneally administered before the onset of ventilation. **(A)** Cell count and **(B)** protein concentration were performed in BAL fluid. **(C)** Lung W/D ratio was measured as an index of pulmonary edema. **(D)** Pulmonary vascular permeability was analyzed by using Evans blue-labeled albumin extravasation into the lung tissue. **(E)** The left lower lung was used for histological evaluation by H&E staining. Original magnification, × 200. Scale bar = 100 μm. **(F)** The severity of lung injury was scored to quantify the severity of lung pathology. Data are expressed as means ± SEM (n = 7). ** p < 0.01.

As shown in Figure 2, DRD1 siRNA, but not DRD2 siRNA, entirely blocked the protective effects of dopamine against mechanical ventilation-induced lung vascular hyperpermeability and lung injury. To further confirm these findings, we examined the role of dopamine in DRD1-defcient mice. The results showed that the inhibitory effects of dopamine on mechanical ventilation-induced increases in BAL cell count, BAL protein levels, lung W/D, Evans blue dye leakage and lung injury score were completely abolished in the absence of DRD1 (Figure 3).

**Figure 4:**
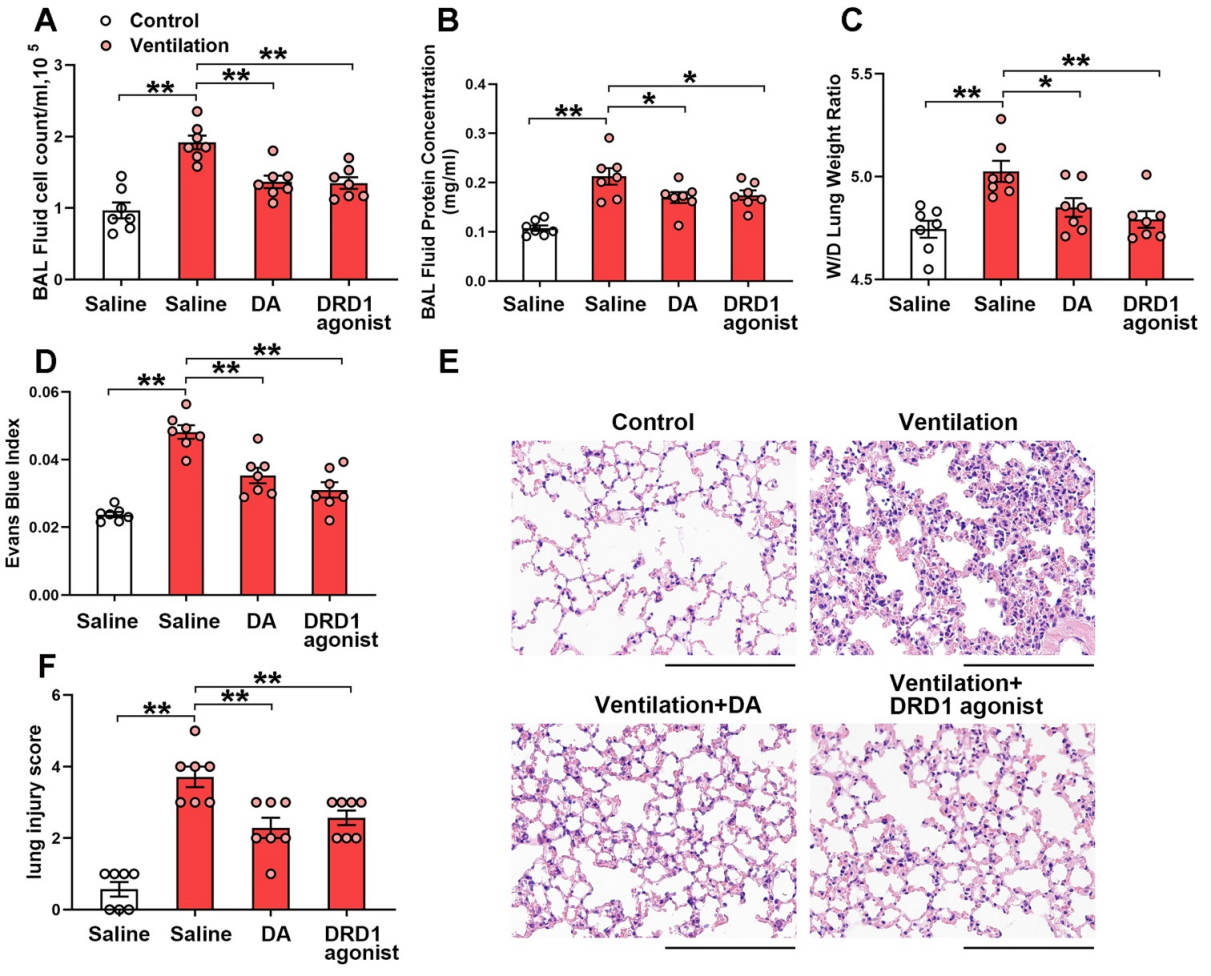
** DRD1 agonist attenuates mechanical ventilation-induced acute lung injury.** Mice were subjected to mechanical ventilation (30 mL/kg) for 4 h. DRD1 agonist SKF-38393 (10 mg/kg) was intraperitoneally administered before the onset of ventilation.** (A)** Cell count and **(B)** protein concentration were performed in BAL fluid. **(C)** Lung W/D ratio was measured as an index of pulmonary edema. **(D)** Pulmonary vascular permeability was analyzed by using Evans blue-labeled albumin extravasation into the lung tissue. **(E)** The left lower lung was used for histological evaluation by H&E staining. Original magnification, × 200. Scale bar = 100 μm. **(F)** The severity of lung injury was scored to quantify the severity of lung pathology. Data are expressed as means ± SEM (n = 7). *p < 0.05, **p < 0.01.

We next investigated the effect of the DRD1-specific agonist SKF-38393 and the DRD2-specific agonist quinpirole on mechanical ventilation-induced lung injury. Both SKF-38393 and quinpirole were intraperitoneally administered before the onset of mechanical ventilation. As shown in Figure 4, prophylactic treatment of SKF-38393 (10 mg/kg) significantly inhibited mechanical ventilation-induced lung vascular hyperpermeability and lung injury, similarly to dopamine. However, all three doses of DRD2 agonist quinpirole (5, 10, 20 mg/kg) had no significant effect on VILI (Figure S7). These findings strongly suggest that dopamine alleviates mechanical ventilation-induced lung vascular hyperpermeability and acute lung injury through DRD1 signaling.

### Prophylactic treatment of dopamine attenuates mechanical stretch-induced α-tubulin deacetylation and subsequent endothelial hyperpermeability via DRD1 signaling

Microtubules play a crucial role in the maintenance of endothelial integrity 8, 9. Stable microtubules undergo diverse post-translational modifications, such as acetylation and detyrosination 12, 13. It has been demonstrated that acetylation of α-tubulin is characteristic of microtubule stabilization 14. Mechanical stretch has been reported to decrease the amount of acetylated microtubule in human pulmonary artery endothelial cells 11. As shown in Figure S8A, we found that mechanical ventilation resulted in a significant decrease in acetylated α-tubulin in lung tissues in a time-dependent manner. Prophylactic administration of the microtubule stabilizer taxol (3.75×10^-7^ mol/kg) completely restored levels of acetylated α-tubulin in ventilated lung tissues (Figure S8B). In addition, mechanical ventilation-induced lung vascular hyperpermeability and lung injury were significantly attenuated following taxol treatment (Figure S8C-H). These results suggest that mechanical stretch-induced deacetylation of microtubule is involved in the development of VILI. In contrast, prophylactic administration of nocodazole (10 mg/kg), a microtubule destabilizer, significantly blocked the protective effects of dopamine against mechanical ventilation-induced lung vascular hyperpermeability and lung injury (Figure S9).

**Figure 5:**
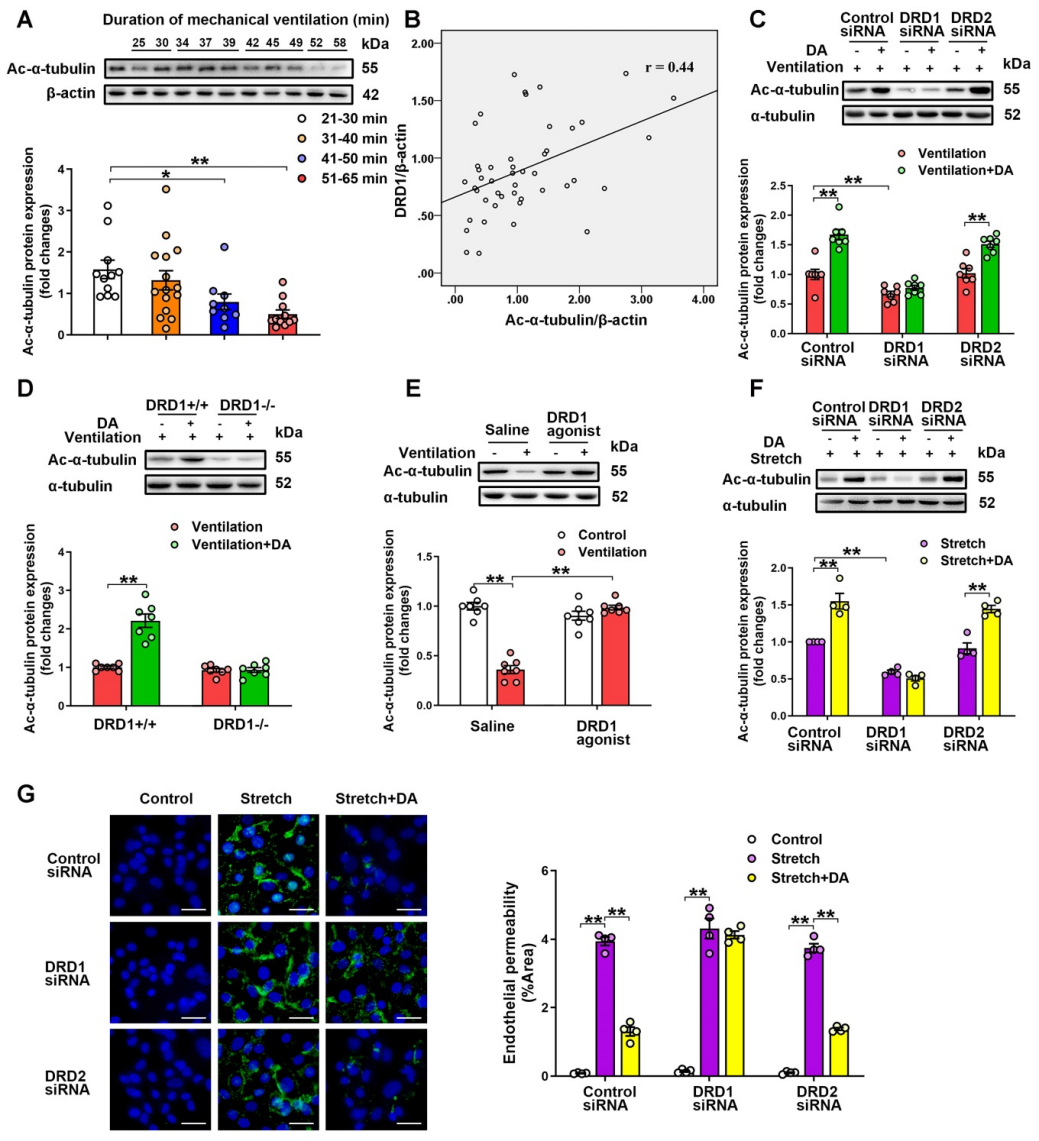
** Dopamine attenuates mechanical stretch-induced α-tubulin deacetylation and subsequent endothelial hyperpermeability via DRD1 signaling. A&B**, Lung tissue were harvested from ventilated patients (n = 11 in 21-30 min group, n = 15 in 31-40 min group, n = 9 in 41-50 min group, n = 11 in 51-65 min group). **(A)** Protein expression of Ac-α-tubulin in the lung homogenates was determined by Western blot analysis and the representative protein bands were presented on the top of corresponding histograms. **(B)** The correlation between DRD1 expression and Ac-α-tubulin expression.** C**, Control or DRD1/DRD2 siRNA (2 mg/kg) were intratracheally instilled seventy-two hours before the onset of ventilation. Dopamine (50 mg/kg) was intraperitoneally administered before the onset of ventilation (n = 7). **D**, Wide-type (DRD1^+/+^) or DRD1 knockout (DRD1^-/-^) mice were subjected to mechanical ventilation (30 mL/kg) for 4 h. Dopamine (50 mg/kg) was intraperitoneally administered before the onset of ventilation (n = 7). **E**, DRD1 agonist SKF-38393 (10 mg/kg) was intraperitoneally administered before the onset of ventilation (n = 7). **C-E**, Protein expression of Ac-α-tubulin in the lung homogenates was determined by Western blot analysis and the representative protein bands were presented on the top of corresponding histograms. Data are expressed as means ± SEM. **F&G**, Primary cultured MLVECs were transfected with siRNA against DRD1/DRD2 (50 nM). Twenty-four hours later, the culture medium was changed, and the cells were subjected to cyclic stretch for 4 h with or without dopamine (0.2 mM) pretreatment. **(F)** Protein expression of Ac-α-tubulin in MLVECs was determined by Western blot analysis and the representative protein bands were presented on the top of corresponding histograms. **(G)** Primary cultured MLVECs were seeded on Collagen I coated Bioflex® culture plates and the FITC fluorescence was detected as described in Materials and Methods. FITC fluorescence signal was visualized by fluorescence microscopy and quantified by using ImageJ. Original magnification, × 200. Scale bar = 20 μm. Data are expressed as means ± SEM (n = 4). *p < 0.05, **p < 0.01.

We then examined the role of dopamine and its receptors in the regulation of α-tubulin acetylation. Using the same samples indicated in Figure 1A, we found that patients who received longer ventilation (mechanical ventilation > 41 min) had significantly lower acetylated α-tubulin levels in lung tissues compared with those who received shorter ventilation (mechanical ventilation < 40 min) (Figure 5A). In addition, the pulmonary levels of acetylated α-tubulin negatively correlated with the duration of mechanical ventilation, and positively correlated with protein levels of DRD1 (Figure 5B). In the murine model of mechanical ventilation-induced lung injury, prophylactic treatment of dopamine abolished mechanical ventilation-induced α-tubulin deacetylation in lung tissues (Figure 5C). In addition, knockdown or deficiency of DRD1, but not knockdown of DRD2, blocked the inhibitory effect of dopamine on mechanical ventilation-induced α-tubulin deacetylation in lung tissues (Figure 5C-D). Moreover, prophylactic treatment of the DRD1 agonist SKF-38393 (10 mg/kg) significantly inhibited mechanical ventilation-induced α-tubulin deacetylation, similarly to dopamine (Figure 5E). However, all three doses of the DRD2 agonist quinpirole (5, 10, 20 mg/kg) had no significant effect on the acetylation of α-tubulin (Figure S10). These findings suggest that prophylactic treatment of dopamine alleviates mechanical ventilation-induced α-tubulin deacetylation via DRD1 signaling.

Preservation of α-tubulin acetylation has been implicated in maintenance of endothelial barrier integrity in lung microvascular endothelial cells 14. Therefore, we next investigated whether dopamine affected cyclic stretch-induced endothelial hyperpermeability by regulating acetylation of α-tubulin in primary cultured MLVECs. As shown in Figure S11A, exposure of MLVECs to 4-h cyclic stretch resulted in a profound decrease in the amount of acetylated microtubules. Consistent with the results of the *in vivo* experiments, pretreatment of cultured MLVECs with dopamine (0.2 mM) significantly attenuated cyclic stretch-induced α-tubulin deacetylation (Figure 5F). In addition, knockdown of DRD1, but not knockdown of DRD2, blocked the inhibitory effect of dopamine on the cyclic stretch-induced α-tubulin deacetylation in endothelial cells.

Next, the effect of dopamine on cyclic stretch-induced endothelial barrier hyperpermeability was tested by visualizing endothelial permeability for macromolecules, i.e. FITC-labeled avidin in MLVEC monolayers. Exposure of MLVECs to 4-h cyclic stretch resulted in marked accumulation of FITC-labeled avidin, indicating increased leakiness in endothelial monolayer (Figure 5G). Dopamine pretreatment significantly decreased the cyclic stretch-induced MLVEC monolayer hyperpermeability for FITC-labeled avidin. Knockdown of DRD1, but not knockdown of DRD2, blocked the protective effect of dopamine against cyclic stretch-induced MLVEC monolayer hyperpermeability. By using a xCELLigence system, we measured transendothelial electrical resistance (TEER) to further observe the effect of dopamine on cyclic stretch-injured MLVEC monolayer integrity. As shown in Figure S12, TEER measurements were taken at 15-min intervals over 20 h to monitor the changes in resistance. We found that cyclic stretch-treated MLVECs exhibited decreased barrier resistance over the course of the experiments, as compared to non-stretched MLVECs. Dopamine pretreatment significantly rescued barrier integrity of cyclic stretch-treated MLVECs, allowing TEER in stretched MLVEC monolayers close to the level of non-stretched MLVECs. In DRD1 siRNA-treated cells, cyclic stretch caused a loss of resistance that persisted in the presence of dopamine. These results suggest that dopamine pretreatment attenuates mechanical stretch-induced α-tubulin deacetylation and subsequent endothelial hyperpermeability via DRD1 signaling.

### DRD1 signaling attenuates cyclic stretch-induced α-tubulin deacetylation and subsequent endothelial hyperpermeability via cAMP/EPAC-mediated inactivation of HDAC6

Previous studies have identified SIRT2 and HDAC6 as α-tubulin deacetylases 15, 16. As shown in Figure S11, the HDAC6 inhibitor tubacin (100 nM), but not the SIRT2 inhibitor AGK-2 (5 μM), blocked cyclic stretch-induced deacetylation of α-tubulin and endothelial hyperpermeability, suggesting a critical role for HDAC6 in cyclic stretch-induced α-tubulin deacetylation. Destabilization of the microtubule network has been shown to result in actin stress fiber formation and contraction in pulmonary endothelial cells, leading to endothelial barrier disruption and increased permeability 8, 9. In addition, previous study has reported that mechanical stretch causes endothelial-mesenchymal transition (EndMT) 5, which is characterized by re-organization of stress fiber and the acquisition of a myofibroblast-like morphology and contractile phenotype 27, 28. We then observed the effect of HDAC6 inhibitor on mechanical stretch-induced stress fiber accumulation and EndMT. As shown in Figure S13A, cyclic stretch resulted in increased stress fiber formation and F-actin reorientation in MLVECs, which were blocked by pretreatment of HDAC6 inhibitor tubacin. Our previous study has shown that 24-h cyclic stretch is able to induce EndMT in MLVECs 5. In the present study, double immunofluorescence staining revealed that 24-h cyclic stretch led to a significant loss of the endothelial marker CD31, accompanying by an increase of the mesenchymal marker α-SMA in MLVECs, both of which were largely prevented by tubacin pretreatment (Figure S13B). In addition, western blot analysis demonstrated that 24-h cyclic stretch-induced acquisition of the mesenchymal markers α-SMA and vimentin was dramatically decreased, whereas the loss of the endothelial markers CD31 and VE-cadherin was largely prevented, by pretreatment of the HDAC6 inhibitor tubacin (Figure S13C). These results provide evidence indicate that HDAC6 inhibition-mediated stabilization of microtubules decreases actin stress fiber formation and EndMT in pulmonary endothelial cells.

**Figure 6:**
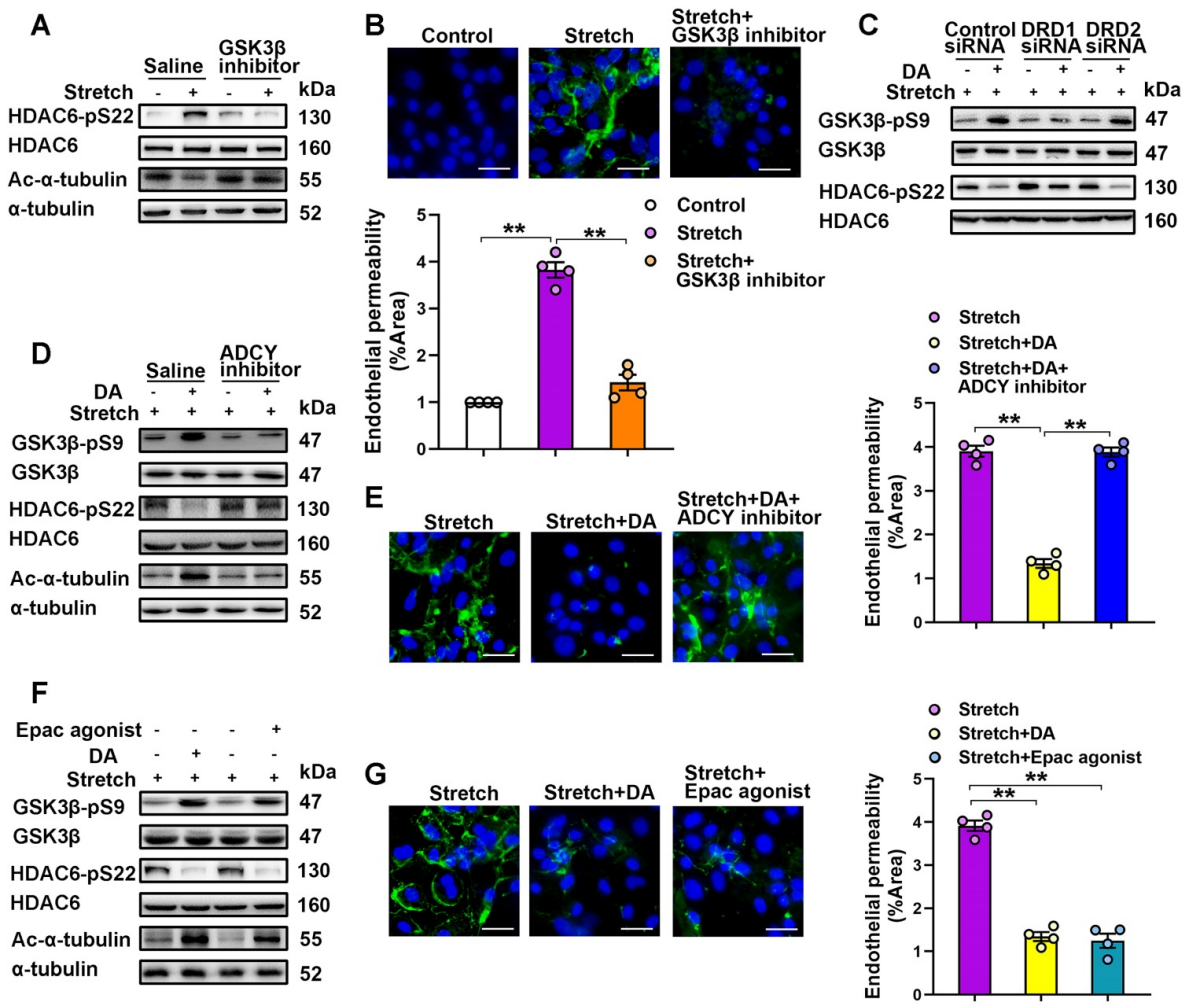
** DRD1 signaling attenuates cyclic stretch-induced α-tubulin deacetylation and subsequent endothelial hyperpermeability via cAMP/EPAC-mediated inactivation of HDAC6. A&B**, Primary cultured MLVECs were pretreated with GSK-3β inhibitor SB216763 (20 μM) and then subjected to cyclic stretch for 4 h.** (A)** Protein expression of HDAC6-pS22 and Ac-α-tubulin in MLVECs was determined by Western blot analysis. **(B)** Primary cultured MLVECs were seeded on Collagen I coated Bioflex® culture plates and the FITC fluorescence was detected as described in Materials and Methods. FITC fluorescence signal was visualized by fluorescence microscopy and quantified by using ImageJ. Original magnification, × 200. Scale bar = 20 μm. **(C)** Primary cultured MLVECs were transfected with siRNA against DRD1/DRD2 (50 nM). Twenty-four hours later, the culture medium was changed, and the cells were subjected to cyclic stretch for 4 h with or without dopamine (0.2 mM) pretreatment. Protein expression of GSK-3β-pS9 and HDAC6-pS22 in MLVECs was determined by Western blot analysis. **D&E**, Primary cultured MLVECs were pretreated with ADCY inhibitor KH7 (5 μM) and then subjected to cyclic stretch for 4 h with or without dopamine (0.2 mM) pretreatment. **(D)** Protein expression of GSK-3β-pS9, HDAC6-pS22 and Ac-α-tubulin in MLVECs was determined by Western blot analysis. **(E)** Primary cultured MLVECs were seeded on Collagen I coated Bioflex® culture plates and the FITC fluorescence was detected as described in Materials and Methods. FITC fluorescence signal was visualized by fluorescence microscopy and quantified by using ImageJ. Original magnification, × 200. Scale bar = 20 μm. **F&G**, Primary cultured MLVECs were pretreated with EPAC agonist 8-pCPT-2′-O-Me-cAMP (100 μM) and then subjected to cyclic stretch for 4 h with or without dopamine (0.2 mM) pretreatment. **(F)** Protein expression of GSK-3β-pS9, HDAC6-pS22 and Ac-α-tubulin in MLVECs was determined by Western blot analysis. **(G)** Primary cultured MLVECs were seeded on Collagen I coated Bioflex® culture plates and the FITC fluorescence was detected as described in Materials and Methods. FITC fluorescence signal was visualized by fluorescence microscopy and quantified by using ImageJ. Original magnification, × 200. Scale bar = 20 μm. Data are expressed as means ± SEM (n = 4). **p < 0.01.

Glycogen-synthase-kinase-3β (GSK-3β) has been found to mediate activation of HDAC6 through phosphorylation at serine 22 in lung microvascular endothelial cells 14. We found that cyclic stretch markedly stimulated phosphorylation of HDAC6 at serine 22 and reduced phosphorylation of GSK-3β at serine 9 in MLVECs (Figure S14A). In addition, pretreatment of GSK-3β inhibitor SB216763 (20 μM) entirely abolished the cyclic stretch-induced phosphorylation of HDAC6, deacetylation of α-tubulin as well as endothelial hyperpermeability (Figure 6A-B, Figure S15A). Since the phosphorylation of GSK-3β at serine 9 represents the inactive form of GSK-3β, these findings indicate that cyclic stretch-induced GSK-3β activation leads to phosphorylation and activation of HDAC6, which subsequently results in deacetylation of α-tubulin and endothelial hyperpermeability in MLVECs.

We then investigated how DRD1 signaling attenuates mechanical stretch-induced α-tubulin deacetylation. DRD1 signaling can stimulate the activity of adenylate cyclase (ADCY) and the production of cyclic AMP (cAMP), an important second messenger for numerous biological processes 29, 30. As shown in Figure S14, we found that the increase of the cAMP levels with ADCY activator forskolin (100 μM) attenuated cyclic stretch-induced α-tubulin deacetylation and MLVEC monolayer hyperpermeability, similar to dopamine treatment. Furthermore, both dopamine and forskolin pretreatment abolished cyclic stretch-induced activation of GSK-3β and HDAC6. As shown in Figure 6C and Figure S15B, knockdown of DRD1, but not knockdown of DRD2, abolished the inhibitive effect of dopamine on GSK-3β/HDAC6 activation. Importantly, the inhibitory effects of dopamine on cyclic stretch-induced activation of GSK-3β and HDAC6, as well as α-tubulin deacetylation and MLVEC monolayer hyperpermeability could be blocked by pretreatment of KH7 (5 μM), an ADCY inhibitor (Figure 6D-E, Figure S15C), suggesting that dopamine-induced inhibition of GSK-3β/HDAC6/α-tubulin deacetylation signaling and the subsequent endothelial hyperpermeability is cAMP-dependent.

Protein kinase A (PKA) and exchange protein activated by cAMP (EPAC) are the two known sensors for intracellular cAMP 31. Therefore, we tested whether these proteins were involved in DA-induced inhibition of α-tubulin deacetylation. Inhibition of PKA with H89 (40 μM) had no effect on DA-induced inhibition of cyclic stretch-induced activation of GSK-3β and HDAC6 as well as α-tubulin deacetylation and MLVEC monolayer hyperpermeability (Figure S16). In contrast, EPAC agonist 8-pCPT-2′-O-Me-cAMP (100 μM) had a similar effect as dopamine on attenuating cyclic stretch-induced activation of GSK-3β and HDAC6, as well as α-tubulin deacetylation and MLVEC monolayer hyperpermeability (Figure 6F-G, Figure S15D). These results suggest that EPAC, but not PKA, is the downstream effector of cAMP that attenuates cyclic stretch-induced GSK-3β/HDAC6/α-tubulin deacetylation signaling and subsequent endothelial hyperpermeability.

Thus, DRD1 signaling attenuates cyclic stretch-induced a-tubulin deacetylation and subsequent endothelial hyperpermeability through cAMP/EPAC-mediated inactivation of HDAC6.

### DRD1 signaling attenuates mechanical stretch-induced α-tubulin deacetylation and subsequent lung endothelial barrier dysfunction via cAMP/EPAC-mediated inactivation of HDAC6* in vivo*

**Figure 7:**
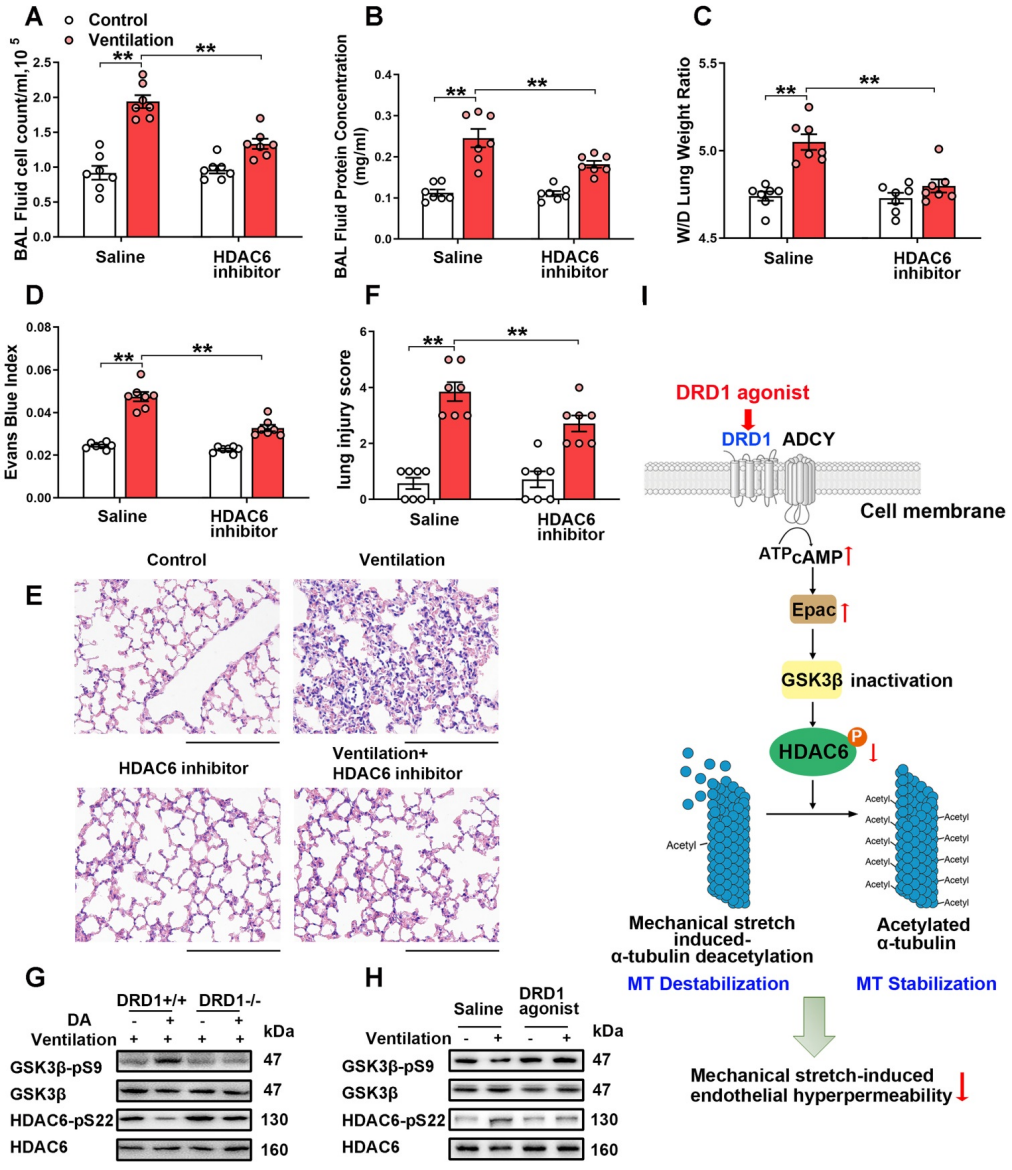
** DRD1 signaling attenuates mechanical stretch-induced α-tubulin deacetylation and subsequent lung endothelial barrier dysfunction via cAMP/EPAC-mediated inactivation of HDAC6 *in vivo*. A-F**, Mice were subjected to mechanical ventilation (30 mL/kg) for 4 h. HDAC6 inhibitor tubacin (1 mg/kg) was intraperitoneally administered before the onset of ventilation. **(A)** Cell count and **(B)** protein concentration were performed in BAL fluid. **(C)** Lung W/D ratio was measured as an index of pulmonary edema. **(D)** Pulmonary vascular permeability was analyzed by using Evans blue-labeled albumin extravasation into the lung tissue. **(E)** The left lower lung was used for histological evaluation by H&E staining. Original magnification, ×200. Scale bar = 100 μm. **(F)** The severity of lung injury was scored to quantify the severity of lung pathology. **G**, Wide-type (DRD1^+/+^) and DRD1 knockout (DRD1^-/-^) mice were subjected to mechanical ventilation (30 mL/kg) for 4 h. Dopamine (50 mg/kg) was intraperitoneally administered before the onset of ventilation. Protein expression of GSK-3β-pS9 and HDAC6-pS22 in lung homogenates was determined by Western blot analysis. **H**, Mice were subjected to mechanical ventilation (30 mL/kg) for 4 h. DRD1 agonist SKF-38393 (10 mg/kg) was intraperitoneally administered before the onset of ventilation. Protein expression of GSK-3β-pS9 and HDAC6-pS22 in lung homogenates was determined by Western blot analysis. **I**, Schematic diagram of the mechanism by which DRD1 signaling protects against lung endothelial barrier dysfunction. Upon activation, DRD1 inactivates GSK-3β and HDAC6 through a cAMP/EPAC-dependent pathway. Inactivation of HDAC6 results in attenuation of mechanical stretch-induced α-tubulin deacetylation and subsequent endothelial hyperpermeability. Data are expressed as means ± SEM (n = 7). **p < 0.01.

We then examined the role of HDAC6 in mechanical ventilation-induced α-tubulin deacetylation and lung vascular hyperpermeability* in vivo*. As shown in Figure S17A, prophylactic administration of the HDAC6 inhibitor tubacin (1 mg/kg) not only increased α-tubulin acetylation in lung tissues of non-ventilated mice, but also significantly alleviated mechanical ventilation-induced α-tubulin deacetylation. In addition, tubacin pretreatment significantly decreased mechanical ventilation-induced increases in BAL cell count and protein levels, lung W/D and Evans blue dye leakage, as well as lung injury scores (Figure 7A-F). Collectively, our findings strongly indicate that activation of HDAC6 is involved in mechanical ventilation-induced α-tubulin deacetylation and lung vascular hyperpermeability *in vivo.*

We then investigated whether and how dopamine treatment affected mechanical ventilation-induced HDAC6 activation in lung tissues. As shown in Figure 7G and Figure S17B, mice subjected to mechanical ventilation for 4 h exhibited activation of GSK-3β and HDAC6 in lung tissues, which was significantly inhibited by prophylactic administration of dopamine. Dopamine-induced inhibition of mechanical ventilation-induced GSK-3β/HDAC6 activation was abolished in DRD1-deficient mice. In addition, the DRD1-specific agonist SKF-38393 (10 mg/kg), but not the DRD2-specific agonist quinpirole (5~20 mg/kg), significantly inhibited mechanical ventilation-induced GSK-3β/HDAC6 activation (Figure 7H and Figure S18). These results suggest that dopamine inhibits mechanical ventilation-induced GSK-3β/HDAC6 activation via DRD1 signaling *in vivo*.

Our *in vitro* study demonstrated that DRD1 signaling might attenuate cyclic stretch-induced α-tubulin deacetylation through cAMP/EPAC-mediated inactivation of HDAC6. To confirm this finding *in vivo*, mice subjected to mechanical ventilation were prophylactically treated with the ADCY inhibitor KH7 (5 μmol/kg) or the EPAC agonist 8-pCPT-2′-O-Me-cAMP (1 mg/kg). As shown in Figure S19, the inhibitory effect of dopamine on mechanical ventilation-induced GSK-3β/HDAC6 activation and α-tubulin deacetylation were blocked by KH7. EPAC agonist treatment had a similar effect to dopamine in attenuating mechanical ventilation-induced GSK-3β/HDAC6 activation and α-tubulin deacetylation (Figure S20).

These observations, together with the results demonstrated in the previous sections indicated that DRD1 signaling might attenuate mechanical stretch-induced α-tubulin deacetylation and subsequent lung endothelial barrier dysfunction through cAMP/EPAC-mediated inactivation of HDAC6 (Figure 7I).

## Discussion

Recent studies have reported the dysregulation of dopamine receptor expression in primary lung tumors and lung tumor cell lines 32-34. However, whether dopamine receptor expression is associated with nontumor-related lung diseases remains largely unknown. In this study, we demonstrated for the first time that DRD1, but not DRD2 and dopamine synthetases, was downregulated in mouse lung tissues exposed to mechanical ventilation. These findings were corroborated by studies involving patients with intraoperative mechanical ventilation support. Furthermore, the pulmonary DRD1 expression levels were negatively correlated with the duration of mechanical ventilation. These initial observations suggest a putative role for DRD1 in the context of VILI.

In the present study, all patients with pulmonary nodules were anesthetized and received physiological ventilation at a low-tidal-volume of 6-8 mL/kg. As for the ventilated animal model, adult mice were anesthetized and ventilated at a high-tidal-volume of 30 mL/kg. Interestingly, both mechanical ventilation strategies led to similar inhibitory effects on pulmonary DRD1 expression. There are two possible reasons for this observation. First, of all patients, about 61% cases have a smoking history. Cigarette smoking has long been associated with local oxidative stress and inflammation in the lung 35. Second, all patients underwent pulmonary lobectomy before collection of lung tissues. Accumulating studies have shown that surgical trauma triggers the systemic stress response characterised by sterile inflammation preceding metabolic and neuroendocrine dysregulation 36, 37. All these factors, including smoking associated oxidative stress and inflammation as well as surgical stress response, may exacerbate the pulmonary changes in response to mechanical stretch.

Notably, Haak et al have found that DRD1 is preferentially expressed in fibroblasts relative to endothelial cells and epithelial cells in the lung 38. This study demonstrates that agonism of DRD1 shifts the phenotype of pulmonary fibroblasts from profibrotic to fibrosis resolving, reversing *in vivo* tissue fibrosis induced by bleomycin. In the present study, double immunofluorescence staining in human and mouse lung tissue sections revealed that DRD1 was co-localized with vascular endothelial cell marker CD31, epithelial cell marker E-cadherin and fibroblast marker PDGFRα. In addition, by using FACS-sorted cell populations, we confirmed the expression of DRD1 in endothelial cells, epithelial cells and fibroblasts in healthy adult lungs of mice. Similar to the findings of Haak et al 38, our results showed that DRD1 expression in fibroblasts was profoundly higher than those in endothelial and epithelial cells. In healthy lung tissues, the immunofluorescence staining indicated that both endothelial cells and epithelial cells were far more widely distributed than fibroblasts. Previous and our own studies have demonstrated the important roles of dopamine-DRD1 pathways in the maintenance of endothelial and epithelial barrier functions in the lung 21, 22. On the other hand, lung fibrotic disease is characterized by proliferation and activation of fibroblasts. We have previous reported that mechanical stretch can induce endothelial-mesenchymal transition and pulmonary fibrosis 5. Future studies are warranted to explore the role of dopamine/DRDs signaling pathways in the pathogenesis of mechanical stretch-induced fibroblast accumulation and lung fibrosis.

Activation of dopamine receptors has been found to reduce acute lung injury of different etiologies. For example, DRD1 mediates dopamine-stimulated edema removal from rat lungs 39. DRD1 agonist not only alleviates endotoxin-induced inflammation and edema in lung tissues 40, but also inhibits NLRP3 inflammasome activation and protects rats from spinal cord injury-induced ALI 41. Moreover, dopamine acts through DRD2 to inhibit pulmonary edema-associated vascular permeability, which conveys protective effects in an endotoxin-induced ALI model 42. In this study, using multiple techniques including intra-pulmonary administration of siRNA targeting dopamine receptors, a DRD1 knockout mouse model and specific dopamine receptor agonists, we provided preliminary insights into the function of dopamine-DRD1 signaling in mediating the protective effects of dopamine against VILI.

Endothelial dysfunction is the underlying component of VILI pathology 4, 11. Mechanical stretch can disrupt endothelial adheren junctions and promote endothelial hyperpermeability in lung tissues 11, 43-45. This breakdown of the pulmonary endothelial barrier is associated with increased levels of systemic pro-inflammatory mediators, which further contribute to adverse outcomes of endothelial dysfunction 3, 43, 46, 47. Consequently, preventing vascular endothelial injury induced by mechanical stretch may become a useful strategy to minimize VILI. Previous studies indicated the protective effect of dopamine against VEGF-mediated vascular permeability 42. In recent years, both DRD1 and DRD2 agonists have been shown to prevent the development of vascular hyperpermeability in animal models of ALI 40, 42, ovarian hyperstimulation syndrome 48, and inflammatory bowel disease 49. In this study, both *in vitro* and *in vivo* experiments indicated that dopamine treatment mitigated mechanical stretch-induced lung vascular endothelial hyperpermeability through DRD1 signaling. The protective effect of a specific DRD1 agonist resulted in a reduction of endothelial dysfunction, thus attenuating leukocyte infiltration and pulmonary edema induced following mechanical stretch. These data indicate that DRD1 agonist might become important in the maintenance of pulmonary vascular endothelial barrier and may show clinical benefit in VILI.

A major implication of mechanical ventilation-induced DRD1 downregulation is that DRD1-targeted therapy would have relevance mostly prior to mechanical ventilation, rather than in the setting of established ongoing injury. Our results indicated that prophylactic activation of DRD1-dependent signaling pathway exhibited significant protective effects against mechanical stretch-induced endothelial barrier dysfunction and acute lung injury. Notably, the present study showed that dopamine protected against mechanical stretch-induced endothelial dysfunction via DRD1/cAMP signaling pathway. Both dopamine and specific DRD1 agonist have been reported to induce a rapid (within 30 minutes) increase of cAMP in various cell types 38, 50, 51, which in turn triggers downstream signaling pathways. Based on these findings, we speculate that prophylactic activation of DRD1-dependent signaling pathway may confer benefit when applied within 30 minutes prior to mechanical ventilation. Nevertheless, future studies should consider the clinically important issue of treatment after injury, in terms of both therapeutic effectiveness and time window constraints.

Accumulating studies have shown that mechanical stretch itself can either deform or destroy the glycocalyx, cilia, adhesion junctions and focal adhesion complexes on the endothelial cells, directly compromising the endothelium integrity and causing hyperpermeability 52. On the other hand, vascular endothelial cells contain mechanosensory complexes, which rapidly react to mechanical stretch, trigger the downstream intracellular signaling pathways, and mediate endothelial cell responses 53, 54. Microtubules are key component of the cytoskeleton and play a critical role in maintaining cell morphology and endothelial barrier integrity 8, 9. Previous studies have demonstrated that microtubule stability is required for the biological role of DRD1 in renal epithelial cells 55 and HEK293 cells stably expressing DRD1 56. The present study demonstrated that pulmonary levels of acetylated α-tubulin, an indicator of stable microtubules, was negatively correlated with duration of mechanical ventilation, and positively correlated with protein levels of DRD1. Mechanical stretch-induced lung endothelial cell hyperpermeability was associated with reduced levels of α-tubulin acetylation, which was rescued by dopamine treatment through DRD1 signaling. In addition, the microtubule destabilizer nocodazole significantly blocked the protective effects of dopamine against VILI. Our data suggest that dopamine-DRD1 signaling decreases mechanical stretch-induced lung endothelial hyperpermeability through the restoration of α-tubulin acetylation and resultant microtubule stabilization.

As a G protein-coupled receptor (GPCR) member, DRD1 initiates its biological function through G proteins, and its stimulation leads to activity of ADCY and production of cAMP, which subsequently activates downstream PKA or EPAC signaling pathways 57. Although PKA signaling has been reported to play diverse and complex roles in the regulation of microtubule stability 58, we did not observe any roles of PKA in dopamine-mediated inhibitory effects on mechanical stretch-induced α-tubulin deacetylation. In contrast, our *in vitro* and* in vivo* experiments indicated that EPAC agonist mimicked, whereas ADCY inhibitor blocked the inhibitory effect of dopamine on mechanical stretch-induced HDAC6 activation, α-tubulin deacetylation and subsequent endothelial hyperpermeability.

Elevated cAMP is known to activate AMPK in various cell types and tissues 59-61. Previous studies have shown that activation of AMPK exerts vascular protective effects and prevents acute lung injury of diverse etiologies, including endotoxin 62-64, acrolein 65 and intestinal ischemia reperfusion 66. In addition, Bone et al report that DRD1 activation results in a robust activation of AMPK in macrophages and alveolar epithelial cells, which provides a substantial anti-inflammatory and bioenergetic advantage and reduces the severity of endotoxin-induced ALI 40. In the present study, we found that DRD1 activation attenuated mechanical stretch-induced α-tubulin deacetylation and subsequent lung endothelial barrier dysfunction via cAMP/EPAC-mediated inactivation of HDAC6. Notably, recent studies demonstrate a mutual regulation between AMPK and HDAC6. For example, inhibition of HDAC6 protects against acute liver failure by activating AMPK signaling pathway 67. In HeLa cells, AMPK activation results in downregulation of HDAC6, which is accompanied by increased acetylation of α-tubulin 68. Whether AMPK signaling pathway is involved in DRD1-mediated protection against mechanical stretch-induced endothelial dysfunction through mutual regulation with HDAC6 merits further investigation.

HDAC6 is a zinc-dependent HDAC that mainly modulates the acetylation status of non-histone substrates, such as α-tubulin and HSP90 69. As an important molecular chaperone, heat shock protein 90 (HSP90) acts as a quality control system, which assists in the maturation of various proteins, crucial mediators of essential functions 70, 71. Recent studies demonstrate that Hsp90 inhibition triggers the activities of the unfolded protein response (UPR) and strong anti-inflammatory responses in the endothelium. These molecular mechanisms may contribute to the vascular protective effects of HSP90 inhibitors in acute lung injury 70, 72, 73. HDAC6 has been found to promote cell proliferation and migration by enhancing HSP90 chaperone function in a variety of cancer cells. Moreover, recent studies designed selective dual inhibitors of HDAC6 and HSP90 as a potentially effective strategy to target lung cancer 71, 74. In LPS-induced lung injury model, general HDAC inhibitors have been found to acetylate and suppress Hsp90 chaperone function, meanwhile provide protection against LPS-induced pulmonary endothelial hyperpermeability 75. Whether dual HDAC6/HSP90 inhibition may provide a better therapeutic strategy for acute lung injury awaits future experiments.

Collectively, this study demonstrated that DRD1 was down-regulated in lung tissues obtained from both patients and experimental animals exposed to mechanical ventilation, and mediated the protective effects of prophylactic dopamine treatment against mechanical stretch-induced vascular endothelial hyperpermeability and lung injury. Upon activation, DRD1 signaling attenuated mechanical stretch-induced α-tubulin deacetylation and subsequent lung endothelial barrier dysfunction via cAMP/EPAC-mediated inactivation of HDAC6. These findings indicate that development of therapeutic strategies to specifically target dopamine-DRD1 signaling might prove useful for protection against mechanical stretch-induced lung injury.

## Supplementary Material

Supplementary figures and tables.

## Abbreviations

VILI: ventilator-induced lung injury
DRD1: Dopamine D1 receptor
DRD2: Dopamine D2 receptor
ALI: acute lung injury
TH: tyrosine hydroxylase
L-DOPA: 3, 4-dihydroxy-l-phenylalanine
DDC: L-DOPA decarboxylase
BAL: bronchoalveolar lavage
W/D: wet/dry weight ratio
H&E: hematoxylin-eosin
SIRT2: sirtuin 2
Ac-α-tubulin: acetylated α-tubulin
GSK-3β-pS9: phosphorylated glycogen-synthase-kinase-3β at serine 9
HDAC6-pS22: phosphorylated histone deacetylase 6 at serine 22
ADCY: adenylate cyclase
cAMP: cyclic AMP
EPAC: exchange protein activated by cAMP
MLVECs: mouse lung vascular endothelial cells
siRNA: small interfering RNA
mRNA: messenger RNA
FITC: fluorescein isothiocyanate
DA: dopamine
MT: microtubule
