# Supplementary material for: DRD1 downregulation contributes to mechanical stretch-induced lung endothelial barrier dysfunction
Source: Theranostics. 2021 Jan 1;11(6):2505–21. doi: 10.7150/thno.46192 (PMC7806475; doi:10.7150/thno.46192)
Supplement: Supplementary file 1 — Supplementary figures and tables. [file thnov11p2505s1.pdf]

Online Data Supplement

DRD1 downregulation contributes to mechanical stretch-induced lung endothelial barrier dysfunction

Yan Wang[1, a], Yu-Jian Liu[2, a], Dun-Feng Xu[1, a], Hui Zhang[1, a], Chu-Fan Xu[1], Yan-Fei Mao[1], Zhou Lv[1], Xiao-Yan Zhu[3, *], Lai Jiang[1, *]

[1] Department of Anesthesiology and Surgical Intensive Care Unit, Xinhua Hospital, Shanghai Jiaotong University School of Medicine, Shanghai, 200092, China

[2] School of Kinesiology, The key Laboratory of Exercise and Health Sciences of Ministry of Education, Shanghai University of Sport, Shanghai, 200438, China

[3] Department of Physiology, Navy Medical University, Shanghai, 200433, China.

[a] The authors contributed equally to this work and should be considered as co-first authors.

Supplementary Materials and Methods

Lung Tissue from Patients with Intraoperative Mechanical Ventilation Support

The lung tissues from ventilated patients were subjected to immunoblotting for tyrosine hydroxylase (TH), L-DOPA decarboxylase (DDC), dopamine D1 receptor (DRD1), dopamine D2 receptor (DRD2), acetylated α-tubulin (Ac-α-tubulin) and β-actin. Total β-actin protein levels were detected for sample loading correction and normalization. A bulk preparation of normal placental protein was used as a control.

Mechanical Ventilation

Male Institute of Cancer Research (ICR) mice (7-9 weeks of age) were purchased from SLAC Laboratory animal (Shanghai China) and kept on a standard laboratory animal facility (25 °C,12 h light/dark cycle) with free access to food and water. DRD1 knockout mice were generously provided by Dr. Rongbin Zhou [1] (Institute of Immunology and the CAS Key Laboratory of Innate Immunity and Chronic Disease, University of Science and Technology of China, Hefei, China). All animals were treated in accordance with The Ethics Committee on Experimental Animals of Shanghai Jiaotong University School of Medicine. The mice were anesthetized with intraperitoneal injection of a combination of 70 mg/kg ketamine and 10 mg/kg xylazine and received a tracheotomy with a 20 G intravenous catheter. The mice were then connected to a ventilator (Inspira, Harvard Apparatus Ltd, Boston, MA, USA). Mechanical ventilation was performed at a tidal volume of 30 mL/kg for 4 h, as described previously [2]. Control mice underwent the same surgical procedures but were allowed to breathe spontaneously. At the end of experiment, the mice were enthanized by exsanguination under anesthesia, and blood and lung tissue samples were collected for analyses. The chosen dose of dopamine (50 mg/kg) [1], DRD1 agonist SKF-38393 (10 mg/kg) [3], microtubule stabilizer taxol (3.75×10^{-7} mol/kg) [4], microtubule destabilizer nocodazole (10 mg/kg) [5], HDAC6 inhibitor tubacin (1 mg/kg) [6], ADCY inhibitor KH7 (5 μmol/kg) [7], EPAC agonist 8-pCPT-2′-O-Me-cAMP (1 mg/kg) [8] were based on previous reports. To ensure the effect of DRD2 agonist quinpirole on VILI, we chose three doses at 5 mg/kg, 10 mg/kg and 20 mg/kg. All these chemical reagents were intraperitoneally injected before the onset of ventilation

Bronchoalveolar Lavage

Bronchoalveolar lavage (BAL) was collected by intratracheal administration of 1 ml sterile Hank’s

balanced salt solution for the measurements of cell count and protein concentration, which were conducted as previously described [2].

Lung Wet-to-Dry Weight Ratio

The index of pulmonary edema formation was measured using the Lung Wet-to-Dry Weight Ratio. The lung weight before and after drying were used to calculate Lung Wet-to-Dry Weight Ratio as previously described [2].

Evans Blue Dye Extravasation Assay

Evans blue dye (30 mg/kg) was injected into the external jugular vein 1 h before termination of ventilation to assess vascular leak, according to the protocol described previously [2].

Lung Histopathological Examination

The lung tissues were fixed in 4% paraformaldehyde, and then processed as described previously. For hematoxylin and eosin (H&E) staining, the lung injury score was assessed by two blinded pathologists with expertise in lung pathology and graded as previously reported [2, 9]: 0 = normal tissue; 1 = tiny inflammatory change; 2 = mild to moderate inflammatory changes without marked damage in the lung architecture; 3 = moderate inflammatory injury with thickening of the alveolar septa; 4 = moderate to severe inflammatory injury with formation of nodules or areas of pneumonitis; and 5 = severe inflammatory injury with total obliteration of the field. The mean score was reported per section.

Immunofluorescence Analysis

Paraffin sections (4 μm) of lung tissues were rehydrated, and microwaved in citric acid buffer to retrieve antigens. After incubation with 10% BSA for 1 h, the sections were incubated with primary antibodies. Primary antibodies included: goat anti-DRD1 (1:50; Santa Cruz, sc-31479), mouse anti-DRD2 (1:50; Santa Cruz, sc-5303), mouse anti-TH (1:50; Santa Cruz, sc-25269), mouse anti-DDC (1:50; Santa Cruz, sc-293287), rabbit anti-CD31 (1:100; Proteintech, 11265-1-AP), rabbit anti-E-cadherin (1:50; Proteintech, 20874-1-AP) and rabbit anti-PDGFR-α (1:200; Abcam, ab203491). Secondary antibodies conjugated with FITC and CY3 were purchased from Proteintech. Finally, nuclei were counterstained with 4’6-diamidino-2-phenylindole (DAPI) (Sigma-Aldrich) and images were collected by fluorescence microscopy (BX53; Olympus, Japan).

MLVECs were fixed with 4% paraformaldehyde for 10 min, permeabilized with 0.1% Triton X-100 for 15 min, and incubated with 10% BSA for 1h. Cells were then incubated with primary antibodies included: mouse anti-CD31 (1:100; Abcam, ab24590), rabbit anti-α-SMA (1:100; Abcam, ab5694). Secondary antibodies conjugated with FITC and CY3 were purchased from Proteintech. Finally, nuclei were counterstained with DAPI (Sigma-Aldrich) and images were collected by fluorescence microscopy (BX53; Olympus, Japan).

Isolation of Mouse Lung Vascular Endothelial Cells (MLVECs)

MLVECs were isolated from Male ICR mice (3-4 weeks) using a modified method as previously described [2, 9, 10]. Briefly, the right ventricle of anesthetized mice was perfused with Dulbecco's Modified Eagle Medium (DMEM, Gibco, Grand Island) to remove blood in the lungs. Peripheral, subpleural pulmonary tissue was cut into pieces and cultured in DMEM with 20% fetal bovine serum(FBS, Gibco, Grand Island) under a 5% CO_2 (37 ℃) atmosphere for 60 h. The diced tissue was then removed, and the adherent cells were cultured in 10% FBS. MLVECs passaged three or four times were used in experiments.

Cell Treatments

After seeded onto collagen I-coated Bioflex® six-well culture plates, cells were treated with various reagents. The doses of all the chemical reagents were chosen according to previous reports and our preliminary experiments. To block the effect of dopamine, ADCY inhibitor KH7 (5 μM) [1] and PKA inhibitor H89 (40 μM) [1] were used before dopamine pretreatment. The agonist including dopamine (0.2 mM) [1], EPAC agonist 8-pCPT-2′-O-Me-cAMP [11], and ADCY activator forskolin (100 μM) [1] were used before cycle stretch. GSK-3β inhibitor SB216763 (20 μM) [12], SIRT2 inhibitor AGK2 (5 μM) [13], HDAC6 inhibitor tubacin (100 nM) [14] were used before cycle stretch. After stretch, supernatants were collected and stored at -70°C for determination and cells were harvested for RNA isolation or protein sample preparation or fixed for staining.

Cyclic stretch

For cyclic stretch, MLVECs were seeded onto collagen I-coated Bioflex® six-well culture plates at density 5×10^5 cells per well and cultured for 48 h to reach confluence. MLVECs were then exposed to high-magnitude [2] (20% linear elongation, sinusoidal wave, 30 cycles/min) for 4 h using the

Flexcell® FX-5000 Tension System, as previously described. The cells that did not receive cyclic stretch were placed in the same incubator next to cyclic stretched cells. The cells and culture supernatants were collected after mechanical stretch for analyses.

Filamentous (F)-actin labeling

F-actin was stained by TRITC-conjugated phalloidin according to manufacturer's instructions (sigma, FAK100). The cells were fixed with 4% paraformaldehyde for 10 min, permeabilized with 0.1% triton X-100 for 15 min, and incubated with 10% BSA for 1 h. Then the cells were stained with TRITC-conjugated phalloidin. The nuclei were stained with DAPI (Sigma-Aldrich), and images were collected by confocal laser scanning microscopy (LSM700; Carl Zeiss Co., Germany).

Measurement of MLVECs permeability

Endothelial permeability was assessed by express permeability testing assay (XperT), using a previously published technique [15]. The assay is based on high-affinity binding of avidin-conjugated, FITC-labeled tracer to the biotinylated extracellular matrix proteins immobilized on the bottom of culture dishes covered with MLVEC monolayers. Permeability assays were performed in a 25 mm BioFlex loading station. The BioFlex plates were coated with biotinylated gelatin (Sigma-Aldrich, St Louis, MO, USA), and MLVECs seeded at a density of 5×10^5 cells per well and grown for 48 h to reach confluence. After the cyclic stretch, cells were fixed with 3.7% formaldehyde, and FITC-avidin (25 μg/ml) was added to the cultured medium for 3 min, then mounted with DAPI. After washing, the elastic bottoms of the BioFlex plates containing the MLVECs were excised with a scalpel and transferred to a microslide, and FITC-avidin fluorescence was measured with fluorescence microscopy (BX53; Olympus, Japan).

Gene Silencing of DRD1 or DRD2

DRD1, DRD2 small interfering RNA (siRNA), and miR-302a-3p mimic were synthesized by Genepharma Corp. (Shanghai, China). The siRNA sequences were provided in Table S4 according to previous reports [1]. For transfection of small interfering RNA, MLVECs were transfected with 50 nM negative control siRNA or DRD1/DRD2 siRNA for 24 h before the cyclic stretch using the Xfect siRNA transfection reagent (Takara). The miR-302a-3p mimic sequences were provided in Table S4 according to previous reports [16]. MLVEC were transfected with negative control miR or

miR-302a-3p mimic for 24 h by using the Xfect siRNA transfection reagents (Takara) in different doses.

In vivo transfection of mouse lungs with siRNA

In vivo DRD1/DRD2 knockdown in mouse lung tissue was achieved by intratracheal siRNA transfection using 2 mg/kg jetPEI transfection reagent (Illkirch, France), according to a previously published protocol [17]. The dose of siRNA was used according to previous reports and our preliminary experiments [9]. Briefly, siRNA and jetPEI polymer at a nuclear/polymer ratio of 7 were diluted with an equal volume of 5% glucose of 20-30 μl working solution. Mice were anesthetized, intubated with a 22 G catheter, and administered 20-30 μl of siRNA working solution. After 72 h, mice were challenged with vehicle or ventilation. The siRNA sequences were provided in Table S4 according to previous reports [1]. The siRNA molecules were synthesized by Genapharma Corp. (Shanghai, China).

Cell sorting

FACS was assessed using a previously published technique [18]. Fully anaesthetized normal or siRNA treated mouse lungs were perfused with cold PBS and the lungs were finely minced with a razor blade in a 100 mm petri dish in 1 ml of cold DMEM medium containing 0.2 mg/ml Liberase DL (Roche) and 100 U/ml DNase I (Roche). Then transferred to 50 ml tubes and incubated at 37 °C for 30 min in a water bath. Enzymatic digestion was inactivated by 10% FBS. The cell suspension was passed once through a 40 μm cell strainer (Fisher), centrifuged at 1300 rpm at 4°C for 10 min and then resuspended in 0.2 ml of FACS buffer (1%BSA, 0.5 μM EDTA pH 7.4 in PBS). The single cell suspension was then incubated with anti-CD140a-APC (1:200, Biolegend, 135908), anti-CD45-PerCp-Cy5.5(1:200, Biolegend, 103132), anti-CD31-PE (1:200, Biolegend, 102408), anti-EPCAM-FITC (1:200, Biolegend, 118207) and DAPI (1:1000) for 30 min on ice. After incubation, cells were washed with ice-cold FACS buffer and resuspended in 1 ml of FACS buffer. FACS sorting was conducted using a BD FACS Aria III (BD Biosciences) gating shown in Figure S2. FACS-sorted epithelial cells, endothelial cells and fibroblasts were collected in TRIzol Reagent (Invitrogen, Carlsbad, CA), and then subjected to mRNA extraction, complementary DNA synthesis and RT– PCR analysis.

Measurement of transendothelial electric resistance

Measurements of transendothelial electrical resistance (TER) across confluent MLVECs monolayers were performed using a xCELLigence Real-Time Cell Analyzer (RTCA) system (RTCA-DP version; Roche Diagnostics, Germany). Changes in impedance (represented as “Cell index”) reflect changes in barrier function and permeability [19, 20] BioFlex plates with stretch-preconditioned MLVECs were treated with trypsin, and cells were seeded at 20000 cells per well onto microelectrode-coated wells of an E-Plate 16 (Roche Applied Science). Analysis of transendothelial resistance were monitored at 15 min intervals over 20 h to monitor the changes in monolayer impedance.

Immunoblotting

Snap-frozen lung tissues or MLVECs were homogenized, and incubated in cold RIPA lysis buffer (Beyotime, China). Proteins were resolved by SDS-PAGE and electrophoretically blotted onto a PVDF membrane (Millipore Corp, Bedford, MA). After blocking, immunoblots were incubated with primary antibody at 4 °C overnight. HRP-conjugated IgG secondary antibody was then asdded for 1 h at room temperature. Primary antibodies included: goat anti-DRD1 (1:200; Santa Cruz, sc-31479), mouse anti-DRD2 (1:1000; Proteintech, 55084-1-AP), mouse anti-TH (1:200; Santa Cruz, sc-25269), mouse anti-DDC (1:200; Santa Cruz, sc-293287), mouse anti-vimentin (1:200; Santa Cruz, sc-373717), rabbit anti-α-SMA (1:1000; Abcam, ab5694), rabbit anti-GSK3β (1:200; Santa Cruz, sc-9166), mouse anti-GSK3β-pS9 (1:200; Santa Cruz, sc-373800), mouse anti-Ac-α-tubulin (1:200; Santa Cruz, sc-23950), rabbit anti-α-tubulin (1:1000; Proteintech, 11224-1-AP), rabbit anti-VE-cadherin (1:1000; Abcam, ab33168), rabbit anti-CD31 (1:1000; Proteintech, 11265-1-AP), rabbit anti-HDAC6 (1:1000; CST, 7612), rabbit anti- HDAC6-pS22 (1:500; sigma, SAB4504190). We also used a chemiluminescent protein ladder to demonstrate the presence of bands at the appropriate weight and confirm the protein identities. The entirety of the western blots showing all proteins detected in this study was provided in the Figure S21. The antibody-reactive bands were visualized using an enhanced chemiluminescence Western blotting detection system (Millipore).

Real-Time RT-PCR

Reverse transcription (RT) was performed with 4 μg RNA of total RNA isolated using TRIzol reagent (Invitrogen, Carlsbad, CA). Quantitative PCR was performed with SYBR® Premix Ex Taq (Takara, Japan) and detected in a StepOne Plus (Applied Biosystem, Foster City, CA) apparatus. Endogenous Ct values of β-actin were used as control. The comparative

threshold cycle (Ct) method with arithmetic formulae ($2^{-\Delta\Delta Ct}$) was used to determine the relative quantization of the indicated gene expression. The online bioinformatics program TargetScan (http://www.targetscan.org) was used to identify potential microRNAs (miRs) that might regulate DRD1 expression. Potential DRD1-targeting miRs were shown in Table S2. To evaluate miRs expression, total miRs were extracted with miRNeasy® Mini Kit (Qiagen, Germany) and subsequently reverse transcribed using miScript II RT Kit (Qiagen). Quantitative PCR was operated using miScript SYBR® Green PCR Kit (Qiagen) and detected in a StepOne Plus (Applied Biosystem, Foster City, CA) apparatus, with the small nuclear RNA (snRNA) U6 was used as an internal control. The comparative threshold cycle (Ct) method with arithmetic formulae ($2^{-\Delta\Delta Ct}$) was used to determine the relative quantization of gene expression. The primer sequences used in this study were provided in Table S3.

Statistical Analysis

Statistical analysis was performed using SPSS 19 (SPSS Inc., Chicago, USA). All data are presented as the mean ± SEM. Normal distribution was assessed using the Shapiro-Wilk test. Statistical significance was determined according to sample distribution and homogeneity of variance. Statistical comparisons between two groups were determined using a two-tailed Student's t test. One-way or two-way ANOVA with post hoc LSD tests was performed for comparisons among multiple groups. A value of $p < 0.05$ was considered significant.

References

1. Yan Y, Jiang W, Liu L, Wang X, Ding C, Tian Z, et al. Dopamine controls systemic inflammation through inhibition of NLRP3 inflammasome. Cell. 2015; 160: 62-73.
2. Dong WW, Liu YJ, Lv Z, Mao YF, Wang YW, Zhu XY, et al. Lung endothelial barrier protection by resveratrol involves inhibition of HMGB1 release and HMGB1-induced mitochondrial oxidative damage via an Nrf2-dependent mechanism. Free Radic Biol Med. 2015; 88: 404-416.
3. Cunningham KA, Callahan PM,Appel JB. Dopamine D1 receptor mediation of the discriminative stimulus properties of SKF 38393. Eur J Pharmacol. 1985; 119: 121-5.
4. Birukova AA, Fu P, Xing J, Yakubov B, Cokic I,Birukov KG. Mechanotransduction by GEF-H1 as a novel mechanism of ventilator-induced vascular endothelial permeability. Am J Physiol Lung Cell Mol Physiol. 2010; 298: L837-48.

5. Feng G,Kaplowitz N. Colchicine protects mice from the lethal effect of an agonistic anti-Fas antibody. J Clin Invest. 2000; 105: 329-39.
6. Yuan H, Li H, Yu P, Fan Q, Zhang X, Huang W, et al. Involvement of HDAC6 in ischaemia and reperfusion-induced rat retinal injury. BMC Ophthalmol. 2018; 18: 300.
7. Lee YS, Tresguerres M, Hess K, Marmorstein LY, Levin LR, Buck J, et al. Regulation of anterior chamber drainage by bicarbonate-sensitive soluble adenylyl cyclase in the ciliary body. J Biol Chem. 2011; 286: 41353-8.
8. Ding H, Bai F, Cao H, Xu J, Fang L, Wu J, et al. PDE/cAMP/Epac/C/EBP-β Signaling Cascade Regulates Mitochondria Biogenesis of Tubular Epithelial Cells in Renal Fibrosis. Antioxid Redox Signal. 2018; 29: 637-652.
9. Xu CF, Liu YJ, Wang Y, Mao YF, Xu DF, Dong WW, et al. Downregulation of R-Spondin1 Contributes to Mechanical Stretch-Induced Lung Injury. Crit Care Med. 2019; 47: e587-e596.
10. Lv Z, Wang Y, Liu YJ, Mao YF, Dong WW, Ding ZN, et al. NLRP3 Inflammasome Activation Contributes to Mechanical Stretch-Induced Endothelial-Mesenchymal Transition and Pulmonary Fibrosis. Crit Care Med. 2018; 46: e49-e58.
11. Kang G, Joseph JW, Chepurny OG, Monaco M, Wheeler MB, Bos JL, et al. Epac-selective cAMP analog 8-pCPT-2'-O-Me-cAMP as a stimulus for Ca2+-induced Ca2+ release and exocytosis in pancreatic beta-cells. J Biol Chem. 2003; 278: 8279-85.
12. Tang XL, Wang CN, Zhu XY, Ni X. Rosiglitazone inhibition of calvaria-derived osteoblast differentiation is through both of PPARgamma and GPR40 and GSK3beta-dependent pathway. Mol Cell Endocrinol. 2015; 413: 78-89.
13. Ramakrishnan G, Davaakhuu G, Kaplun L, Chung WC, Rana A, Atfi A, et al. Sirt2 deacetylase is a novel AKT binding partner critical for AKT activation by insulin. J Biol Chem. 2014; 289: 6054-66.
14. Borgas D, Chambers E, Newton J, Ko J, Rivera S, Rounds S, et al. Cigarette Smoke Disrupted Lung Endothelial Barrier Integrity and Increased Susceptibility to Acute Lung Injury via Histone Deacetylase 6. Am J Respir Cell Mol Biol. 2016; 54: 683-96.
15. Dubrovskyi O, Birukova AA,Birukov KG. Measurement of local permeability at subcellular level in cell models of agonist- and ventilator-induced lung injury. Lab Invest. 2013; 93: 254-63.

16. Zhu X, Li H, Wu Y, Zhou J, Yang G, Wang W, et al. CREB-upregulated lncRNA MEG3 promotes hepatic gluconeogenesis by regulating miR-302a-3p-CRTC2 axis. J Cell Biochem. 2019; 120: 4192-4202.

17. Fu P, Usatyuk PV, Lele A, Harijith A, Gregorio CC, Garcia JG, et al. c-Abl mediated tyrosine phosphorylation of paxillin regulates LPS-induced endothelial dysfunction and lung injury. Am J Physiol Lung Cell Mol Physiol. 2015; 308: L1025-38.

18. Schafer MJ, White TA, Iijima K, Haak AJ, Ligresti G, Atkinson EJ, et al. Cellular senescence mediates fibrotic pulmonary disease. Nat Commun. 2017; 8: 14532.

19. Atienza JM, Yu N, Kirstein SL, Xi B, Wang X, Xu X, et al. Dynamic and label-free cell-based assays using the real-time cell electronic sensing system. Assay Drug Dev Technol. 2006; 4: 597-607.

20. Solly K, Wang X, Xu X, Strulovici B, Zheng W. Application of real-time cell electronic sensing (RT-CES) technology to cell-based assays. Assay Drug Dev Technol. 2004; 2: 363-72.

Supplemental tables

Table S1. Characteristics of the Patients

**Characteristics of the patients**	
**Age, yr**	50.06±8.99
**Weight, kg**	67.04±9.3
**Sex, no. (%)**	
F	13(28.26%)
M	33(71.74%)
**Smoking history, no. (%)**	
Y	28(60.87%)
N	18(39.13%)
**ASA physical statue, no. (%)**	
I	31(67.39%)
II	15(32.61%)
**Ventilation time (min), no. (%)**	
21-30	11(23.91%)
31-40	15(32.61%)
41-50	9(19.57%)
51-60	11(23.91%)

Table S2. microRNAs potentially targeting dopamine receptors

Dopamine receptors	microRNAs
**DRD1**	miR-30
	miR-181
	miR-302
**DRD2**	miR-9
	miR-124
	miR-141
	miR-200
**DRD3**	No Conserved Found
**DRD4**	No Conserved Found
**DRD5**	No Conserved Found

Table S3. Primer sequences used in the study

Primer Name	5'-3'
qDRD1(forward)	GTCCAGGGGTTTTGGGAGAA
qDRD1(reverse)	AGTCACTTTTCGGGGATGCT
qDRD2(forward)	AGTGAACAGGCGGAGAATGG
qDRD2(reverse)	TAGACCGTGGTGGGATGGAT
qTH(forward)	TACTTTGTGCGCTTCGAGGT
qTH(reverse)	GGAACCTTGTCCTCTCTGGC
qDDC(forward)	AGTTCGCAGAGCTGGACAAT
qDDC(reverse)	AGGCTCCACATCAGGGTACA
qβ-actin (forward)	CTGTATGCCTCTGGTCGTAC
qβ-actin (reverse)	TGATGTCACGCACGATTTCC
miR-30c-5p (forward)	CGCTGTAAACATCCTACACTCTCAGC
miR-181c-5p (forward)	CGAACATTCAACCTGTCGGTGAGT
miR-302a-3p (forward)	CGCTAAGTGCTTCCATGTTTTGGTGA
U6 (forward)	AGAGAAGATTAGCATGGCCCC

Table S4. microRNA mimic and small interfering RNA sequences used in the study

Sequence Name	5’-3’
miR-302a-3p mimic	UAAGUGCUUCCAUGUUUUGGUGA
miR control	UUGUACUACACAAAAGUACUG
DRD1 siRNA	GGGAGACUAAAGUCCUGAA
DRD2 siRNA	GGCCAUGCCUAUGUUGUAU
Negative control	UCCUCCGAACGUGUCACGUTT

Supplemental figures

Figure S1. Expression of dopamine synthetase and dopamine receptor in mouse pulmonary tissue. (A) Human lung tissues were stained with fluorophore-labeled antibodies against DRD1, DRD2, TH and DDC (CY3, red) and CD31, E-cadherin and PDGFR-α (FITC, green). DAPI stain was used to detect nuclei (blue). Original magnification, × 200. Scale bars correspond to 50 μm. **(B)** Mouse lung tissues were stained with fluorophore-labeled antibodies against DRD1, DRD2, TH and DDC (CY3, red) and CD31, E-cadherin and PDGFR-α (FITC, green). DAPI stain was used to detect nuclei (blue). Original magnification, × 200. Scale bars correspond to 50 μm.

Figure S2. Expression of dopamine synthetase and dopamine receptor in mouse lung cells. (A-D) Gating strategy for isolation of fibroblasts, epithelial and endothelial cells for normal or siRNA-treated mouse lungs. **(A)**Total single-cell suspensions (P1) were gated to exclude doublets (P2) and CD45+ cells (P3). **(B)**Fibroblasts (PDGFR-α+, EPCAM-, CD31- and CD45-), **(C)** epithelial cells (EPCAM+, PDGFR-α-, CD31- and CD45-) and endothelial cells (CD31+, PDGFR-α-, EPCAM- and CD45-) were sorted from the P3 population. **(E-H)** Quantitative real-time reverse-transcription polymerase chain reaction was used to determine mRNA expression of DRD1, DRD2, TH and DDC. Data are expressed as means ± SEM (n = 3).

Figure S3. Expression of dopamine receptor in mouse extra-pulmonary tissues after ventilation. Mice were subjected to mechanical ventilation (30 mL/kg) for 4 h and the heart **(A)**, brain **(B)** and kidney **(C)** were harvested after ventilation. Protein expression of DRD1 and DRD2 in these tissues was determined by Western blot analysis and the representative protein bands are presented on the top of corresponding histograms. Data are expressed as means ± SEM (n = 7).

Figure S4. Mechanical ventilation results in a significant increase of microRNA(miR)-302a-3p, which may contribute to the downregulation of DRD1. (A) Mice were subjected to mechanical ventilation (30 mL/kg) for 4 h. Quantitative real-time reverse-transcription polymerase chain reaction was used to determine the expression of miR-30c-5p/181c-5p/302a-3p. Data are expressed as means ± SEM (n = 6). **(B-C)** Primary cultured mouse lung vascular endothelial cells were transfected with miR-302a-3p mimic or control miR. Twenty-four hours later, DRD1 and DRD2 mRNA and protein expression were determined. Data are expressed as means ± SEM (n = 4). * $p < 0.05$, ** $p < 0.01$.

Figure S5. Expression of dopamine receptor in mouse pulmonary and extra-pulmonary tissues after intratracheal DRD1/DRD2 siRNA transfection. Mice were intratracheally instilled with control or DRD1/DRD2 siRNA (2 mg/kg). Seventy-two hours later, the pulmonary, heart, brain and kidney were harvested. Protein expression of DRD1**(A)** and DRD2**(B)** in these tissues was determined by Western blot analysis and the representative protein bands are presented on the left of corresponding histograms. Data are expressed as means ± SEM (n = 7). **$p < 0.01$.

Figure S6. Expression of dopamine receptor in mouse lung cells after intratracheal DRD1/DRD2 siRNA transfection. Mice were intratracheally instilled with control or DRD1/DRD2 siRNA (2 mg/kg). Seventy-two hours later, the pulmonary was harvested for cell sorting. Quantitative real-time reverse-transcription polymerase chain reaction was used to determine the expression of DRD1 and DRD2 in endothelial **(A&C)** and epithelial **(B&D)** cells. Data are expressed as means ± SEM (n = 3). **$p < 0.01$.

Figure S7. DRD2 agonist had no effect on mechanical ventilation-induced acute lung injury. Mice were subjected to mechanical ventilation (30 mL/kg) for 4 h. DRD2 agonist quinpirole (5, 10, 20 mg/kg) was intraperitoneally administered before the onset of ventilation. **(A)** Cell count and **(B)** protein concentration were performed in BAL fluid. **(C)** Lung W/D ratio was measured as an index of pulmonary edema. **(D)** Pulmonary vascular permeability was analyzed by using Evans blue-labeled albumin extravasation into the lung tissue. **(E)** The left lower lung was used for histological evaluation by H&E staining. Original magnification, × 200. Scale bar = 100 μm. **(F)** The severity of lung injury was scored to quantify the severity of lung pathology. Data are expressed as means ± SEM (n = 7). **p < 0.01.

Figure S8. Taxol attenuates mechanical stretch-induced a-tubulin deacetylation and acute lung injury. (A) Mice were subjected to mechanical ventilation (30 mL/kg) and lung tissues were harvested at the time points indicated. Protein expression of Ac-α-tubulin in lung homogenates was determined by Western blot analysis and the representative protein bands are presented on the top of corresponding histograms. **B-H**, Mice were subjected to mechanical ventilation (30 mL/kg) for 4 h. Microtubule stabilizer taxol (3.75×10^{-7} mol/kg) was intraperitoneally administered before the onset of ventilation. **(B)** Protein expression of Ac-α-tubulin in the lung homogenates was determined by Western blot analysis and the representative protein bands were presented on the top of corresponding histograms. **(C)** Cell count and **(D)** protein concentration were performed in BAL fluid. **(E)** Lung W/D ratio was measured as an index of pulmonary edema. **(F)** Pulmonary vascular permeability was analyzed by using Evans blue-labeled albumin extravasation into the lung tissue. **(G)** The left lower lung was used for histological evaluation by H&E staining. Original magnification, × 200. Scale bar = 100 μm. **(H)** The severity of lung injury was scored to quantify the severity of lung pathology. Data are expressed as means ± SEM (n = 7). * p < 0.05, ** p < 0.01.

Figure S9. Nocodazole abrogates the protective effect of dopamine against mechanical ventilation-induced acute lung injury. Mice were intraperitoneally administered with microtubule damaging agent nocodazole (10 mg/kg) and then subjected to mechanical ventilation (30 mL/kg) for 4 h. Dopamine (50 mg/kg) was intraperitoneally administered before the onset of ventilation. **(A)** Cell count and **(B)** protein concentration were performed in BAL fluid. **(C)** Lung W/D ratio was measured as an index of pulmonary edema. **(D)** Pulmonary vascular permeability was analyzed by using Evans blue-labeled albumin extravasation into the lung tissue. **(E)** The left lower lung was used for histological evaluation by H&E staining. Original magnification, × 200. Scale bar = 100 μm. **(F)** The severity of lung injury was scored to quantify the severity of lung pathology. Data are expressed as means ± SEM (n = 7). * p < 0.05, ** p < 0.01.

Figure S10. DRD2 agonist had no effect on mechanical ventilation-induced α-tubulin deacetylation. Mice were subjected to mechanical ventilation (30 mL/kg) for 4 h. DRD2 agonist quinpirole (5, 10, 20 mg/kg) was intraperitoneally administered before the onset of ventilation. Protein expression of Ac-α-tubulin in MLVECs was determined by Western blot analysis and the representative protein bands were presented on the left of corresponding histograms. Data are expressed as means ± SEM (n = 7). ** p < 0.01.

Figure S11. HDAC6 not SIRT2 contributes to cyclic stretch-induced α-tubulin deacetylation and MLVEC monolayer hyperpermeability. Primary cultured MLVECs were pretreated with **(A)** SIRT2 inhibitor AGK2 (5 μM) or **(B)** HDAC6 inhibitor tubacin (100 nM) and then subjected to

cyclic stretch for 4 h. Protein expression of Ac-α-tubulin in MLVECs was determined by Western blot analysis and the representative protein bands were presented on the top of corresponding histograms. **(C)** Primary cultured MLVECs were seeded on Collagen I coated Bioflex® culture plates and the FITC fluorescence was detected as described in Materials and Methods. FITC fluorescence signal was visualized by fluorescence microscopy and quantified by using ImageJ. Original magnification, × 200. Scale bar = 20 μm. Data are expressed as means ± SEM (n = 4). ** p < 0.01.

Figure S12. DRD1 signaling attenuates cyclic stretch-induced endothelial hyperpermeability. Primary cultured MLVECs were transfected with siRNA against DRD1/DRD2 (50 nM). Twenty-four hours later, the culture medium was changed, and the cells were subjected to cyclic stretch for 4 h with or without dopamine (0.2 mM) pretreatment. MLVECs were then replated onto micro electrodes. Transendothelial electrical resistance was monitored over the time. Data are expressed as means ± SEM (n = 4). ** p < 0.01.

Figure S13. HDAC6 inhibitor attenuates mechanical stretch-induced actin cytoskeletal reorganization and EndMT. (A) Primary cultured MLVECs were pretreated with HDAC6 inhibitor tubacin (100 nM) and then subjected to cyclic stretch for 4 h. MLVECs were then stained with TRITC-conjugated–phalloidin. DAPI stain was used to detect nuclei (blue). Original magnification, × 200, scale bars correspond to 50 μm. **(B)** Primary cultured MLVECs were pretreated with HDAC6 inhibitor tubacin (100 nM) and then subjected to cyclic stretch for 24 h. Fluorophore-labeled antibodies against CD31 (CY3, red) and α-SMA (FITC, green). DAPI stain was used to detect nuclei (blue). Original magnification, × 200, scale bars correspond to 50 μm. **(C)**Protein expression of VE-cadherin, CD31, α-SMA and Vimentin in MLVECs was determined by Western blot analysis and the representative protein bands were presented on the top of corresponding histograms. Data are expressed as means ± SEM (n = 4). ** p < 0.01.

Figure S14. ADCY activator forskolin attenuated cyclic stretch-induced α-tubulin deacetylation and MLVEC monolayer hyperpermeability. Primary cultured MLVECs were pretreated with dopamine (0.2 mM) or ADCY activator forskolin (100 μM) and then subjected to cyclic stretch for 4 h. **(A)** Protein expression of GSK-3β-pS9, HDAC6-pS22 and Ac-α-tubulin in MLVECs was determined by Western blot analysis and the representative protein bands were presented on the left of corresponding histograms. **(B)** Primary cultured MLVECs were seeded on Collagen I coated Bioflex® culture plates and the FITC fluorescence was detected as described in Materials and Methods. FITC fluorescence signal was visualized by fluorescence microscopy and quantified by using ImageJ. Original magnification, × 200. Scale bar = 20 μm. Data are expressed as means ± SEM (n = 4). ** p < 0.01.

Figure S15. DRD1 signaling attenuates cyclic stretch-induced α-tubulin deacetylation and subsequent endothelial hyperpermeability via cAMP/EPAC-mediated inactivation of HDAC6 (related to Figure 6). (A) Primary cultured MLVECs were pretreated with GSK-3β inhibitor SB216763 (20 μM) and then subjected to cyclic stretch for 4 h. Protein expression of HDAC6-pS22 and Ac-α-tubulin in MLVECs was determined by Western blot analysis and corresponding histograms was shown. **(B)** Primary cultured MLVECs were transfected with siRNA against DRD1/DRD2 (50 nM). Twenty-four hours later, the culture medium was changed, and the cells were subjected to cyclic stretch for 4hr with or without dopamine (0.2 mM) pretreatment. Protein

expression of GSK-3β-pS9 and HDAC6-pS22 in MLVECs was determined by Western blot analysis and corresponding histograms was shown. **(C)** Primary cultured MLVECs were pretreated with ADCY inhibitor KH7 (5 μM) and then subjected to cyclic stretch for 4hr with or without dopamine (0.2 mM) pretreatment. Protein expression of GSK-3β-pS9, HDAC6-pS22 and Ac-α-tubulin in MLVECs was determined by Western blot analysis and corresponding histograms was shown. **(D)** Primary cultured MLVECs were pretreated with EPAC agonist 8-pCPT-2′-O-Me-cAMP (100 μM) and then subjected to cyclic stretch for 4hr with or without dopamine (0.2 mM) pretreatment. Protein expression of GSK-3β-pS9, HDAC6-pS22 and Ac-α-tubulin in MLVECs was determined by Western blot analysis and corresponding histograms was shown. Data are expressed as means ± SEM (n = 4). **p < 0.01.

Figure S16. The inhibitory effect of dopamine on cyclic stretch-induced α-tubulin deacetylation and MLVEC monolayer hyperpermeability could not be blocked by PKA inhibitor. Primary cultured MLVECs were pretreated with PKA inhibitor H89 (40 μM) and then subjected to cyclic stretch for 4 h with or without dopamine (0.2 mM) pretreatment. **(A)** Protein expression of GSK-3β-pS9, HDAC6-pS22 and Ac-α-tubulin in MLVECs was determined by Western blot analysis and the representative protein bands were presented on the left of corresponding histograms. **(B)** Primary cultured MLVECs were seeded on Collagen I coated Bioflex® culture plates and the FITC fluorescence was detected as described in Materials and Methods. FITC fluorescence signal was visualized by fluorescence microscopy and quantified by using ImageJ. Original magnification, × 200. Scale bar = 20 μm. Data are expressed as means ± SEM (n = 4). ** p < 0.01.

Figure S17. DRD1 signaling attenuates mechanical stretch-induced α-tubulin deacetylation and subsequent lung endothelial barrier dysfunction via cAMP/EPAC-mediated inactivation of HDAC6 in vivo (related to Figure 7). (A) Mice were subjected to mechanical ventilation (30 mL/kg) for 4 h. HDAC6 inhibitor tubacin (1 mg/kg) was intraperitoneally administered before the onset of ventilation. Protein expression of Ac-α-tubulin in MLVECs was determined by Western blot analysis and the representative protein bands were presented on the top of corresponding histograms. **(B)** Wide-type ($DRD1^{+/+}$) and DRD1 knockout ($DRD1^{-/-}$) mice were subjected to mechanical ventilation (30 mL/kg) for 4 h. Dopamine (50 mg/kg) was intraperitoneally administered before the onset of ventilation. Protein expression of GSK-3β-pS9 and HDAC6-pS22 in lung homogenates was determined by Western blot analysis and corresponding histograms was shown. **C**, Mice were subjected to mechanical ventilation (30 mL/kg) for 4 h. DRD1 agonist SKF-38393 (10 mg/kg) was intraperitoneally administered before the onset of ventilation. Protein expression of GSK-3β-pS9 and HDAC6-pS22 in lung homogenates was determined by Western blot analysis and corresponding histograms was shown. Data are expressed as means ± SEM (n = 7). **p < 0.01.

Figure S18. DRD2 agonist had no effect on mechanical ventilation-induced activation of GSK-3β and HDAC6. Mice were subjected to mechanical ventilation (30 mL/kg) for 4 h. DRD2 agonist quinpirole (5, 10, 20 mg/kg) was intraperitoneally administered before the onset of ventilation. Protein expression of GSK-3β-pS9 and HDAC6-pS22 in lung homogenates was determined by Western blot analysis and the representative protein bands were presented on the left of corresponding histograms. Data are expressed as means ± SEM (n = 7). ** p < 0.01.

Figure S19. The inhibitory effect of dopamine on mechanical ventilation-induced GSK-3β/HDAC6 activation and α-tubulin deacetylation could be blocked by ADCY inhibitor.

Mice were intraperitoneally administered with ADCY inhibitor KH7 (5 μmol/kg) and then subjected to mechanical ventilation (30 mL/kg) for 4 h. Dopamine (50 mg/kg) was intraperitoneally administered before the onset of ventilation. Protein expression of GSK-3β-pS9, HDAC6-pS22 and Ac-α-tubulin in the lung homogenates was determined by Western blot analysis and the representative protein bands were presented on the left of corresponding histograms. Data are expressed as means ± SEM (n = 7). ** $p < 0.01$.

Figure S20. The effect of EPAC agonist on mechanical ventilation-induced GSK-3β/HDAC6 activation and α-tubulin deacetylation. Mice were intraperitoneally administered with EPAC agonist 8-pCPT-2′-O-Me-cAMP (1 mg/kg) and then subjected to mechanical ventilation (30 mL/kg) for 4 h. Dopamine (50 mg/kg) was intraperitoneally administered before the onset of ventilation. Protein expression of GSK-3β-pS9, HDAC6-pS22 and Ac-α-tubulin in the lung homogenates was determined by Western blot analysis and the representative protein bands were presented on the left of corresponding histograms. Data are expressed as means ± SEM (n = 7). ** $p < 0.01$.

Figure S21. The entirety of western blot. Lane 1: luminescence marker; Lane 2: histiocyte lysate.

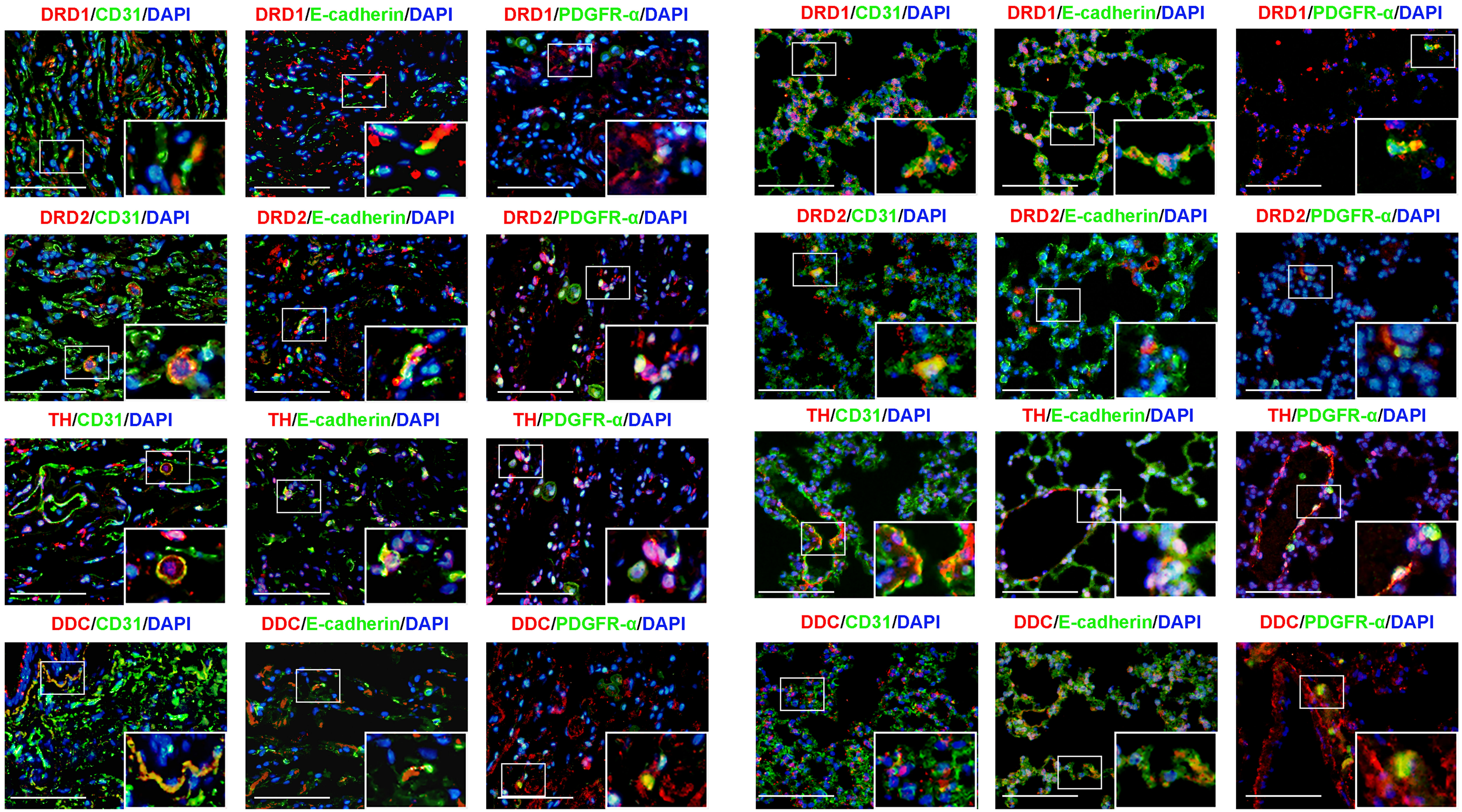
DRD1/CD31/DAPI
DRD1/E-cadherin/DAPI
DRD1/PDGFR-α/DAPI
DRD1/CD31/DAPI
DRD1/E-cadherin/DAPI
DRD1/PDGFR-α/DAPI
DRD2/CD31/DAPI
DRD2/E-cadherin/DAPI
DRD2/PDGFR-α/DAPI
DRD2/CD31/DAPI
DRD2/E-cadherin/DAPI
DRD2/PDGFR-α/DAPI
TH/CD31/DAPI
TH/E-cadherin/DAPI
TH/PDGFR-α/DAPI
TH/CD31/DAPI
TH/E-cadherin/DAPI
TH/PDGFR-α/DAPI
DDC/CD31/DAPI
DDC/E-cadherin/DAPI
DDC/PDGFR-α/DAPI
DDC/CD31/DAPI
DDC/E-cadherin/DAPI
DDC/PDGFR-α/DAPI

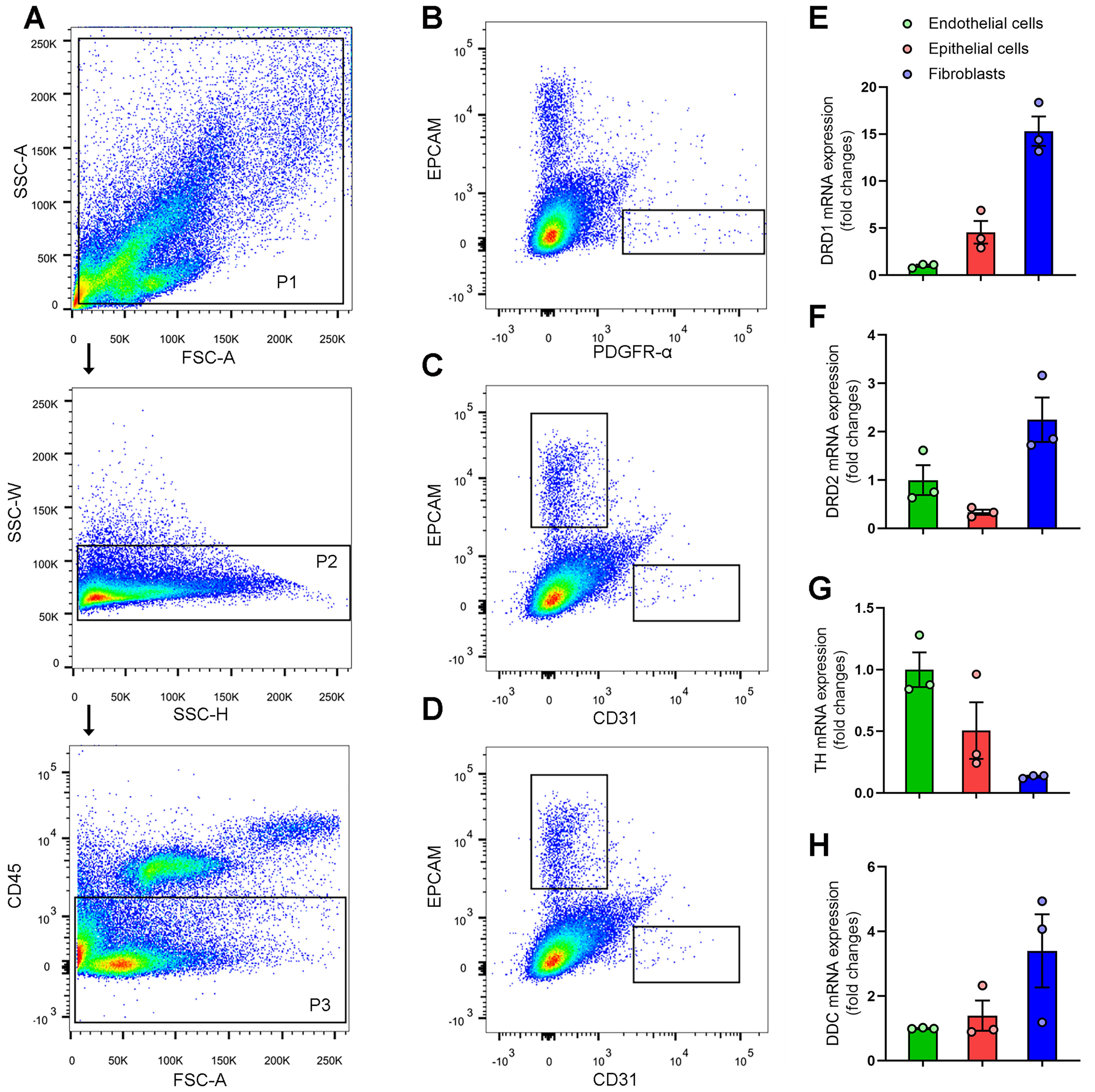

A
SSC-A
FSC-A
P1
SSC-W
SSC-H
P2
CD45
FSC-A
P3
B
EPCAM
PDGFR-α
C
EPCAM
CD31
D
EPCAM
CD31
E
Endothelial cells
Epithelial cells
Fibroblasts
DRD1 mRNA expression (fold changes)
F
DRD2 mRNA expression (fold changes)
G
TH mRNA expression (fold changes)
H
DDC mRNA expression (fold changes)

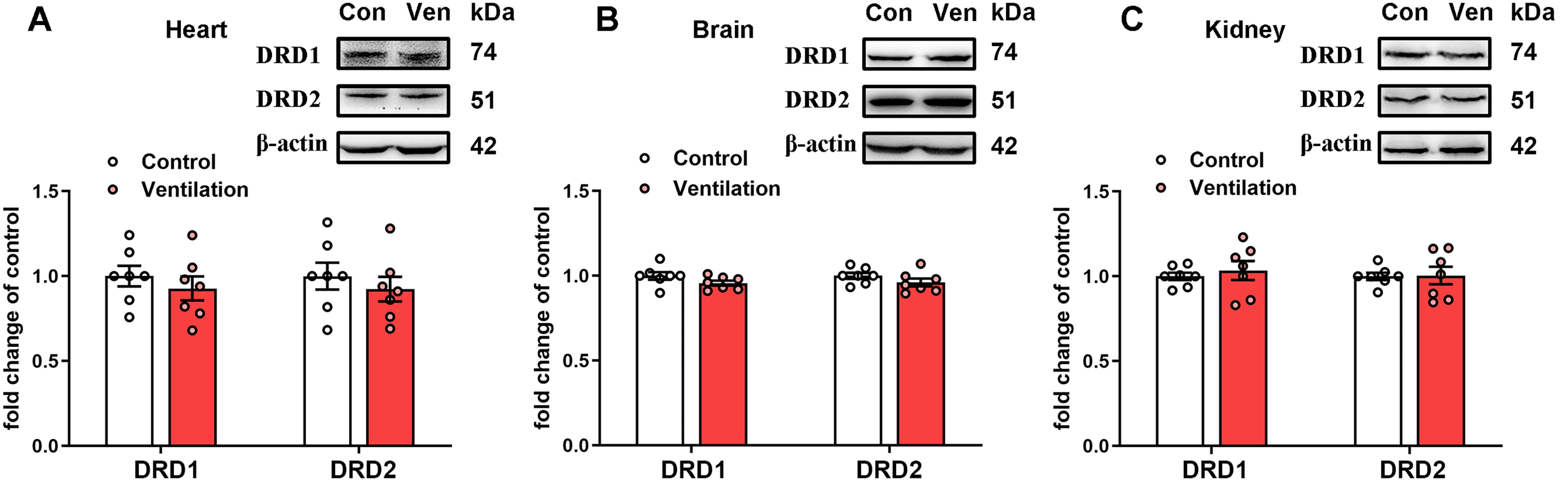
A
Heart
Con
Ven
kDa
DRD1
74
DRD2
51
β-actin
42
Control
Ventilation
fold change of control
1.5
1.0
0.5
0.0
DRD1
DRD2
B
Brain
Con
Ven
kDa
DRD1
74
DRD2
51
β-actin
42
Control
Ventilation
fold change of control
1.5
1.0
0.5
0.0
DRD1
DRD2
C
Kidney
Con
Ven
kDa
DRD1
74
DRD2
51
β-actin
42
Control
Ventilation
fold change of control
1.5
1.0
0.5
0.0
DRD1
DRD2

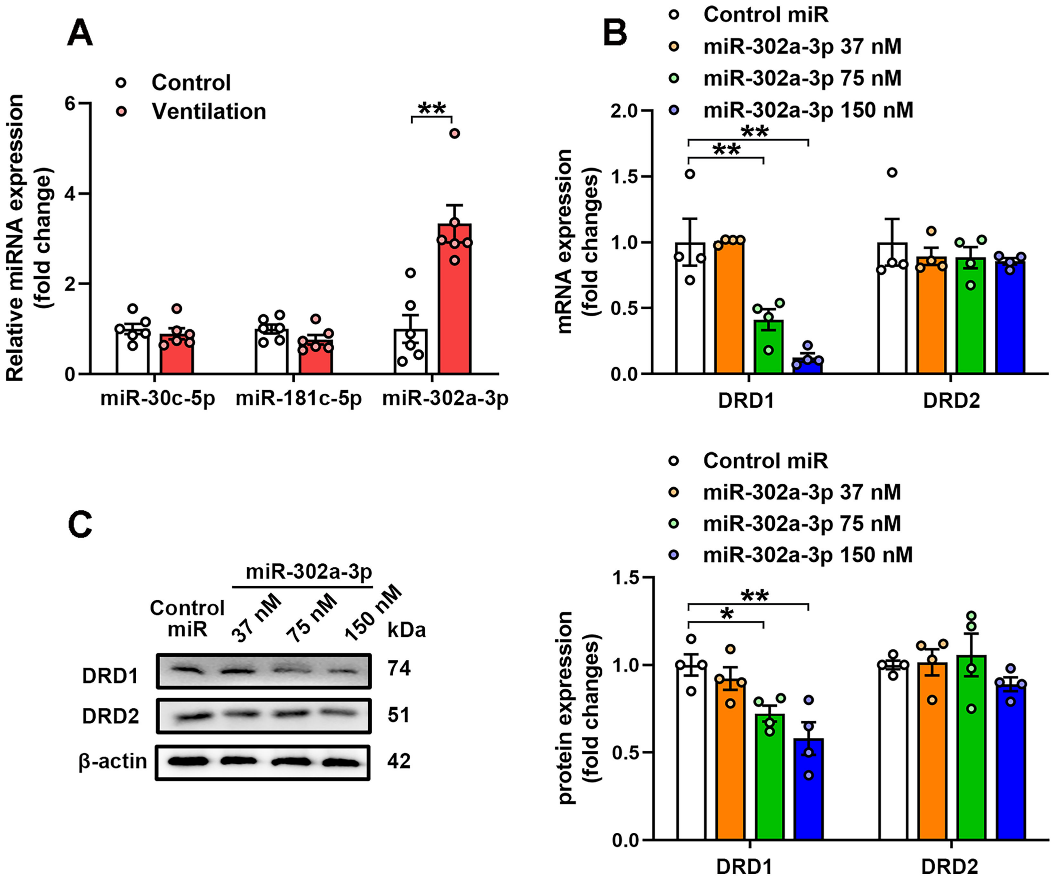

A
Control
Ventilation
Relative miRNA expression (fold change)
**
miR-30c-5p
miR-181c-5p
miR-302a-3p
B
Control miR
miR-302a-3p 37 nM
miR-302a-3p 75 nM
miR-302a-3p 150 nM
mRNA expression (fold changes)
DRD1
DRD2
C
miR-302a-3p
Control miR
37 nM
75 nM
150 nM
kDa
DRD1
DRD2
β-actin
74
51
42
protein expression (fold changes)

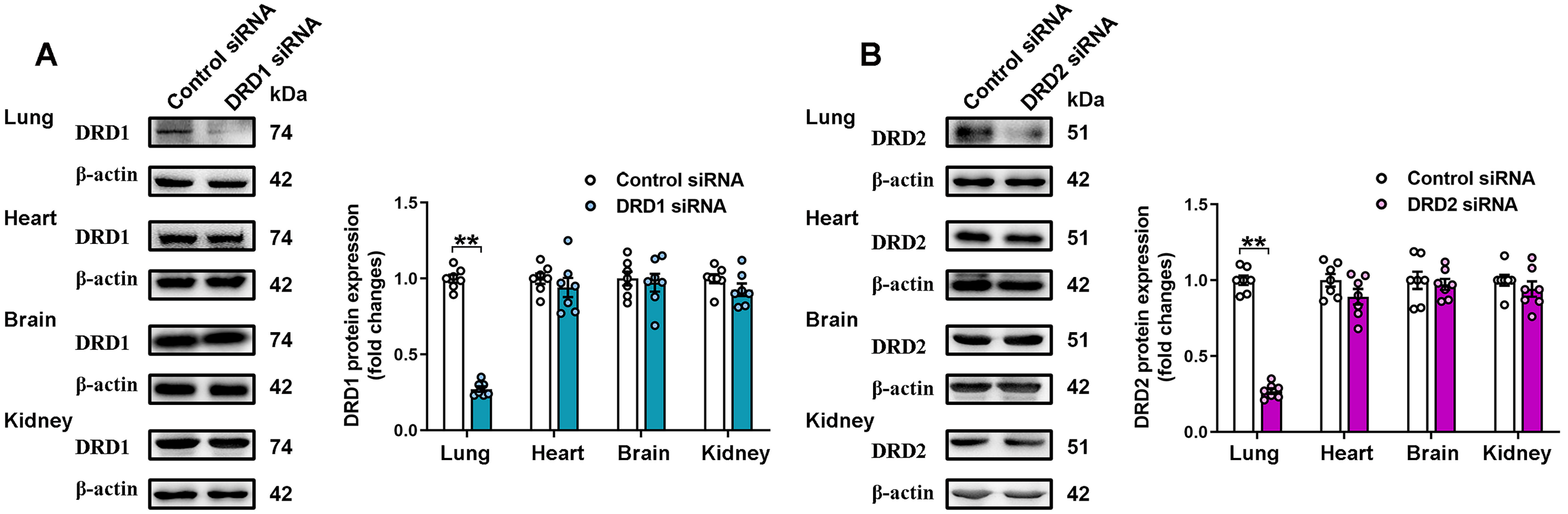
A
Control siRNA
DRD1 siRNA
kDa
Lung
DRD1
74
β-actin
42
Heart
DRD1
74
β-actin
42
Brain
DRD1
74
β-actin
42
Kidney
DRD1
74
β-actin
42
Control siRNA
DRD1 siRNA
DRD1 protein expression (fold changes)
1.5
1.0
0.5
0.0
**
Lung
Heart
Brain
Kidney
B
Control siRNA
DRD2 siRNA
kDa
Lung
DRD2
51
β-actin
42
Heart
DRD2
51
β-actin
42
Brain
DRD2
51
β-actin
42
Kidney
DRD2
51
β-actin
42
Control siRNA
DRD2 siRNA
DRD2 protein expression (fold changes)
1.5
1.0
0.5
0.0
**
Lung
Heart
Brain
Kidney

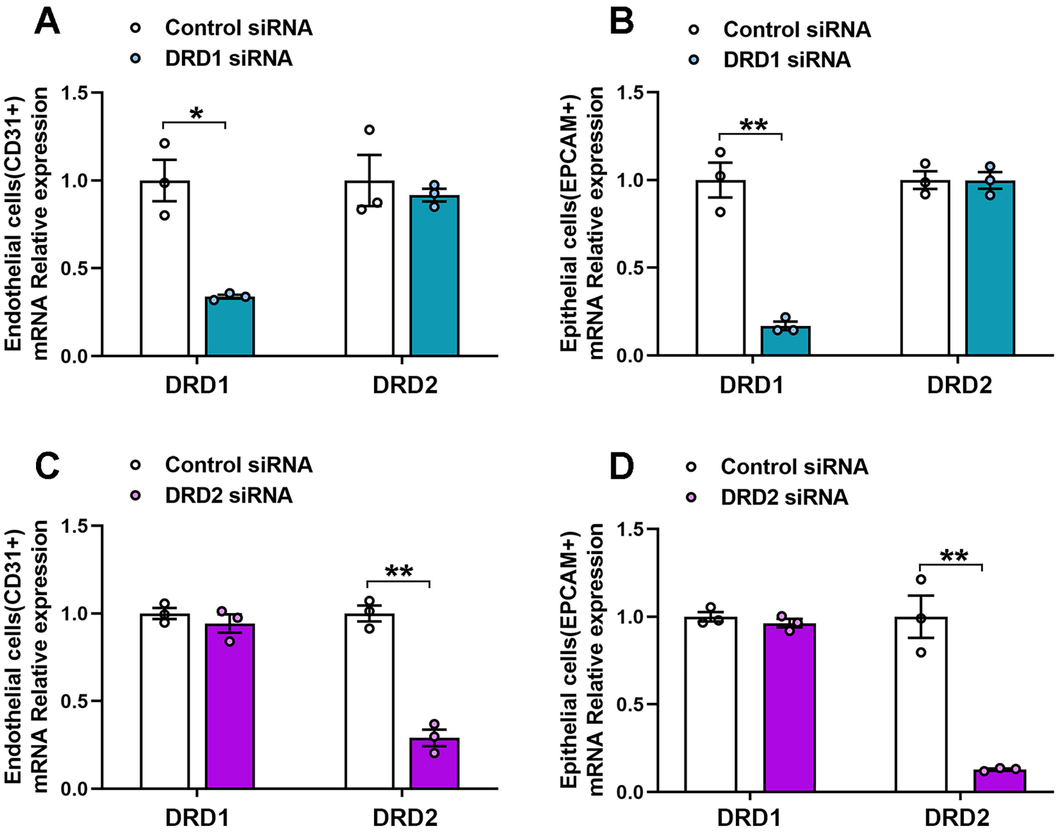

A
Control siRNA
DRD1 siRNA
Endothelial cells(CD31+) mRNA Relative expression
1.5
1.0
0.5
0.0
*
DRD1
DRD2
B
Control siRNA
DRD1 siRNA
Epithelial cells(EPCAM+) mRNA Relative expression
1.5
1.0
0.5
0.0
**
DRD1
DRD2
C
Control siRNA
DRD2 siRNA
Endothelial cells(CD31+) mRNA Relative expression
1.5
1.0
0.5
0.0
**
DRD1
DRD2
D
Control siRNA
DRD2 siRNA
Epithelial cells(EPCAM+) mRNA Relative expression
1.5
1.0
0.5
0.0
**
DRD1
DRD2

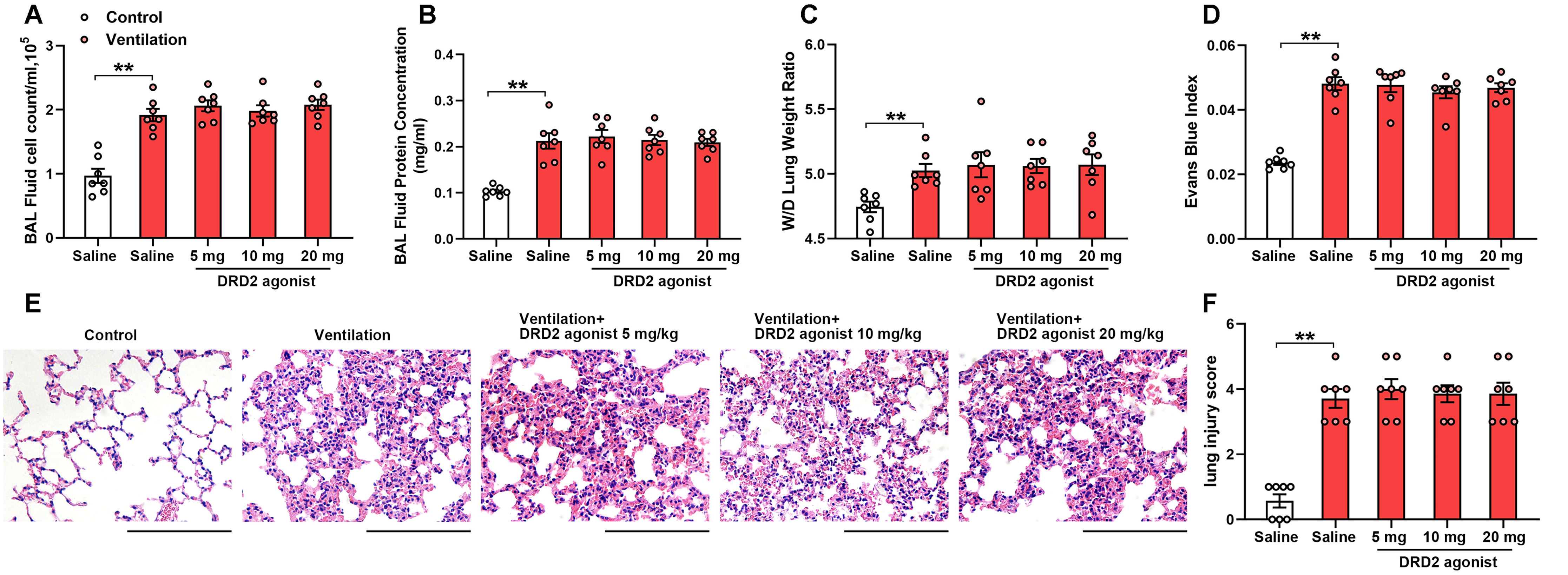
A
Control
Ventilation
BAL Fluid cell count/ml,10^5
**
Saline
Saline
5 mg
10 mg
20 mg
DRD2 agonist
B
BAL Fluid Protein Concentration (mg/ml)
**
Saline
Saline
5 mg
10 mg
20 mg
DRD2 agonist
C
W/D Lung Weight Ratio
**
Saline
Saline
5 mg
10 mg
20 mg
DRD2 agonist
D
Evans Blue Index
**
Saline
Saline
5 mg
10 mg
20 mg
DRD2 agonist
E
Control
Ventilation
Ventilation+ DRD2 agonist 5 mg/kg
Ventilation+ DRD2 agonist 10 mg/kg
Ventilation+ DRD2 agonist 20 mg/kg
F
lung injury score
**
Saline
Saline
5 mg
10 mg
20 mg
DRD2 agonist

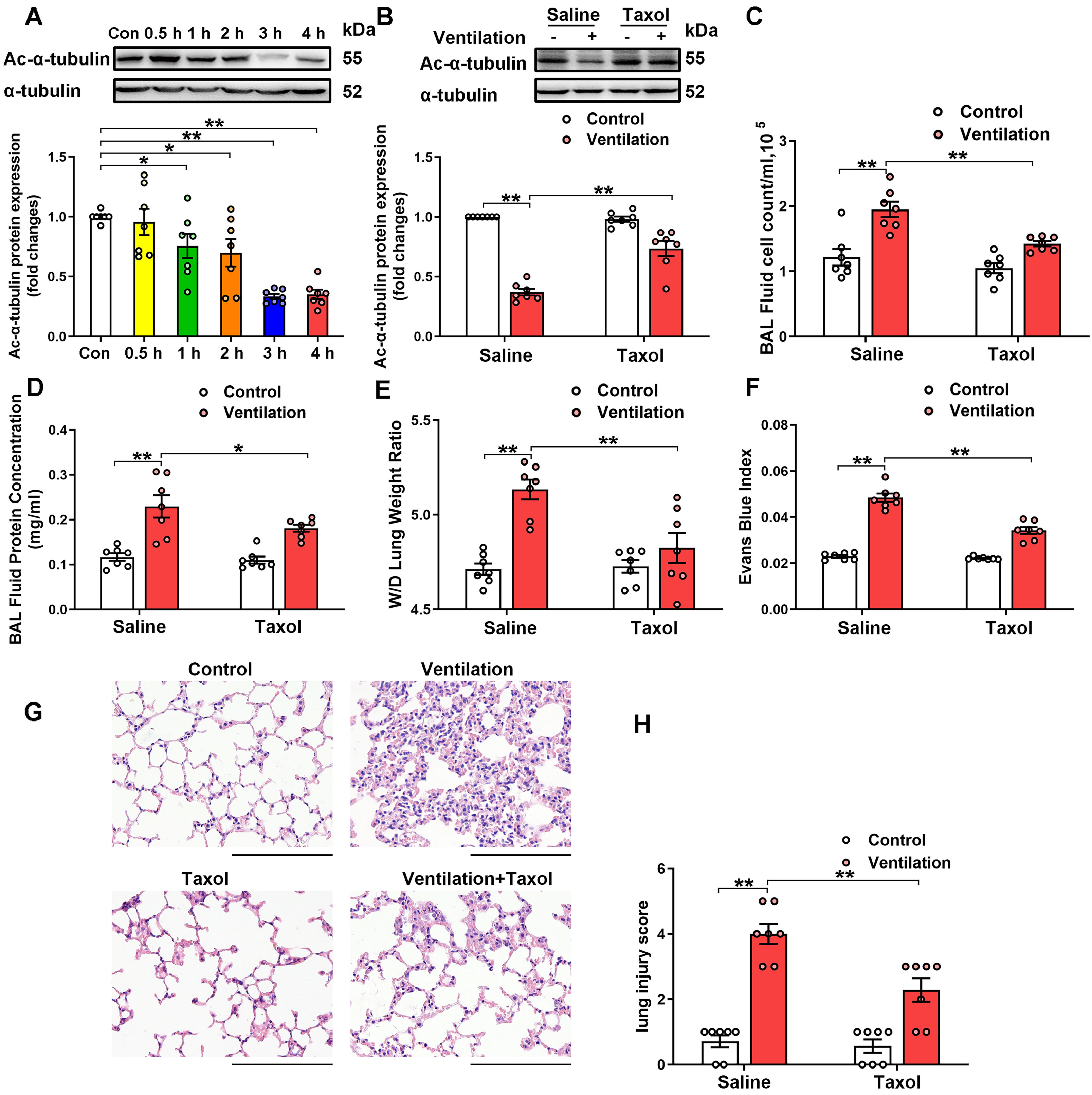
A
Con 0.5 h 1 h 2 h 3 h 4 h
kDa
Ac-α-tubulin
55
α-tubulin
52
Ac-α-tubulin protein expression (fold changes)
Con 0.5 h 1 h 2 h 3 h 4 h
B
Saline
Taxol
Ventilation
Ac-α-tubulin
α-tubulin
Control
Ventilation
Saline
Taxol
C
BAL Fluid cell count/ml,10 5
D
BAL Fluid Protein Concentration (mg/ml)
E
W/D Lung Weight Ratio
F
Evans Blue Index
G
Control
Ventilation
Taxol
Ventilation+Taxol
H
lung injury score

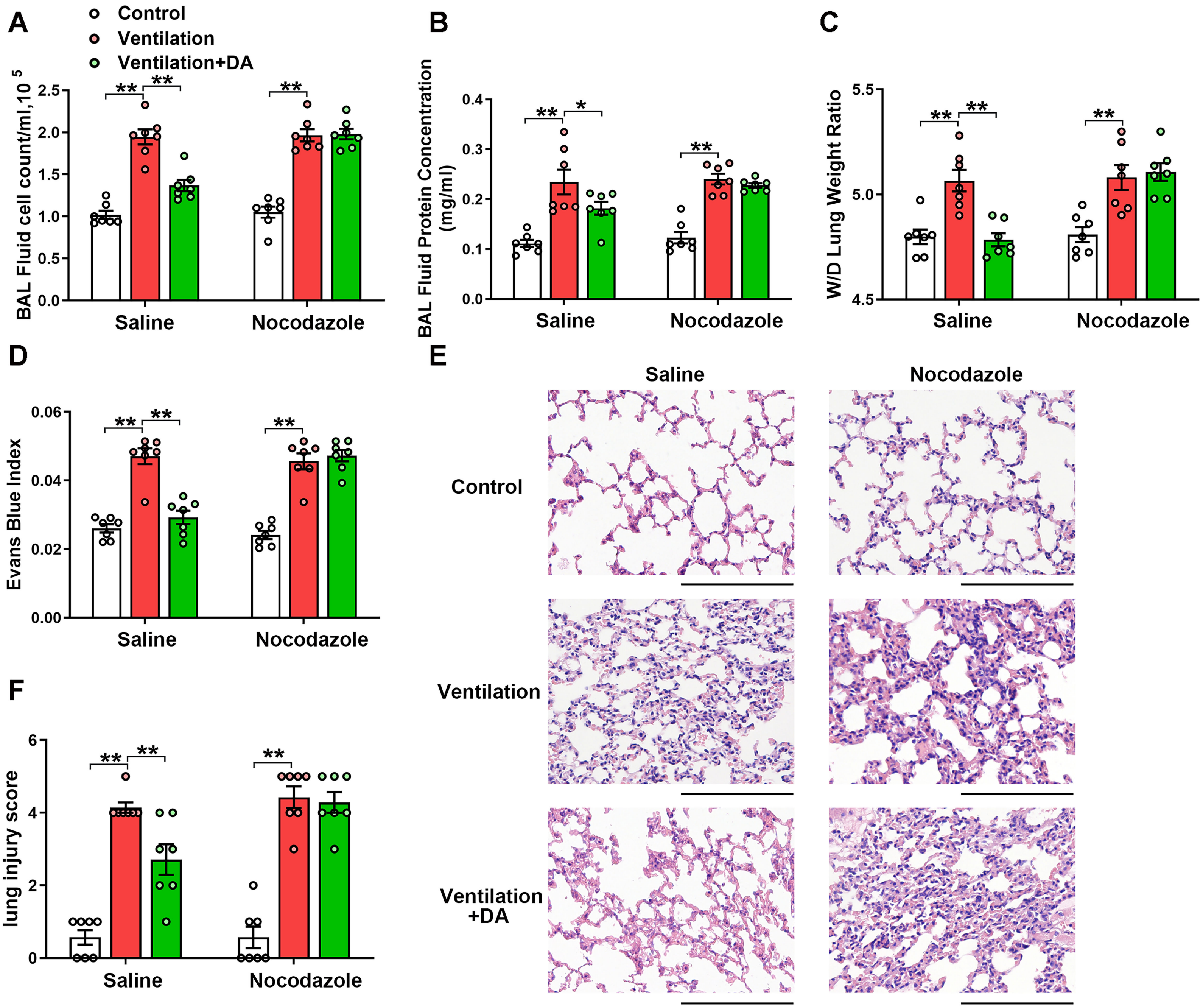
A
Control
Ventilation
Ventilation+DA
BAL Fluid cell count/ml,10 5
Saline
Nocodazole
B
BAL Fluid Protein Concentration (mg/ml)
C
W/D Lung Weight Ratio
D
Evans Blue Index
E
Saline
Nocodazole
Control
Ventilation
Ventilation +DA
F
lung injury score

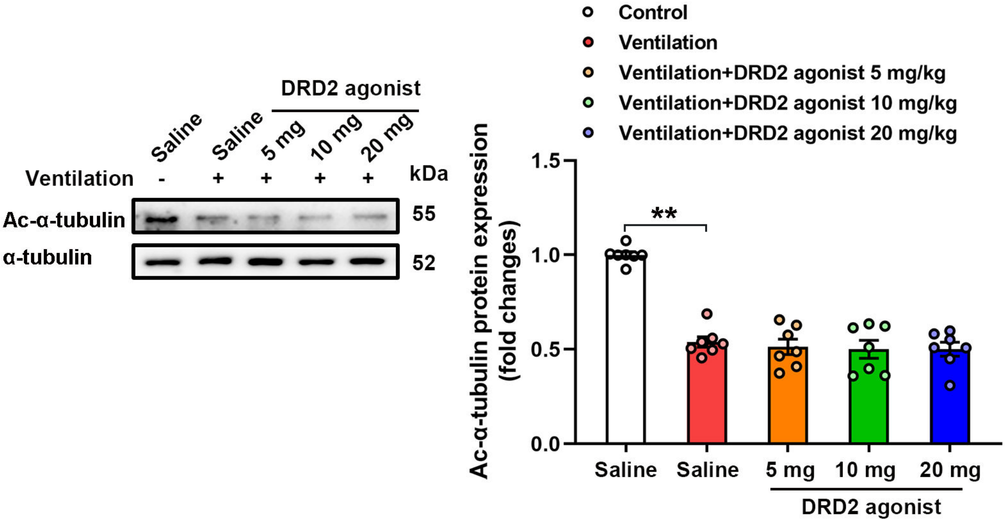

DRD2 agonist
Saline
Saline
5 mg
10 mg
20 mg
Ventilation
-
+
+
+
+
kDa
Ac-α-tubulin
55
α-tubulin
52
Control
Ventilation
Ventilation+DRD2 agonist 5 mg/kg
Ventilation+DRD2 agonist 10 mg/kg
Ventilation+DRD2 agonist 20 mg/kg
Ac-α-tubulin protein expression (fold changes)
1.5
1.0
0.5
0.0
**
Saline
Saline
5 mg
10 mg
20 mg
DRD2 agonist

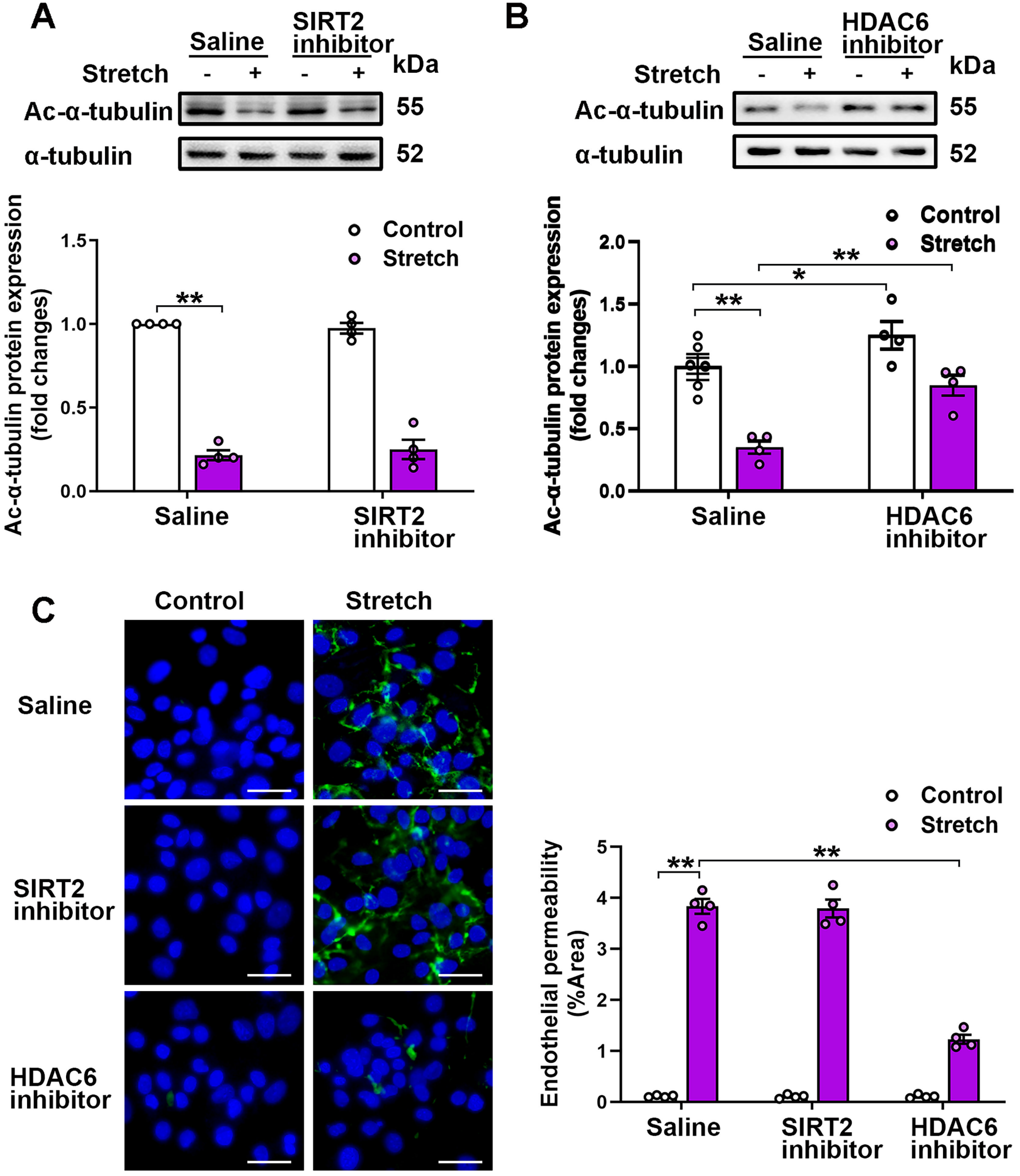
A
Saline
SIRT2 inhibitor
Stretch
-
+
-
+
kDa
Ac-α-tubulin
55
α-tubulin
52
Ac-α-tubulin protein expression (fold changes)
Control
Stretch
**
Saline
SIRT2 inhibitor
B
Saline
HDAC6 inhibitor
Stretch
kDa
Ac-α-tubulin
55
α-tubulin
52
Ac-α-tubulin protein expression (fold changes)
Control
Stretch
**
*
**
Saline
HDAC6 inhibitor
C
Control
Stretch
Saline
SIRT2 inhibitor
HDAC6 inhibitor
Control
Stretch
Endothelial permeability (%Area)
**
**
Saline
SIRT2 inhibitor
HDAC6 inhibitor

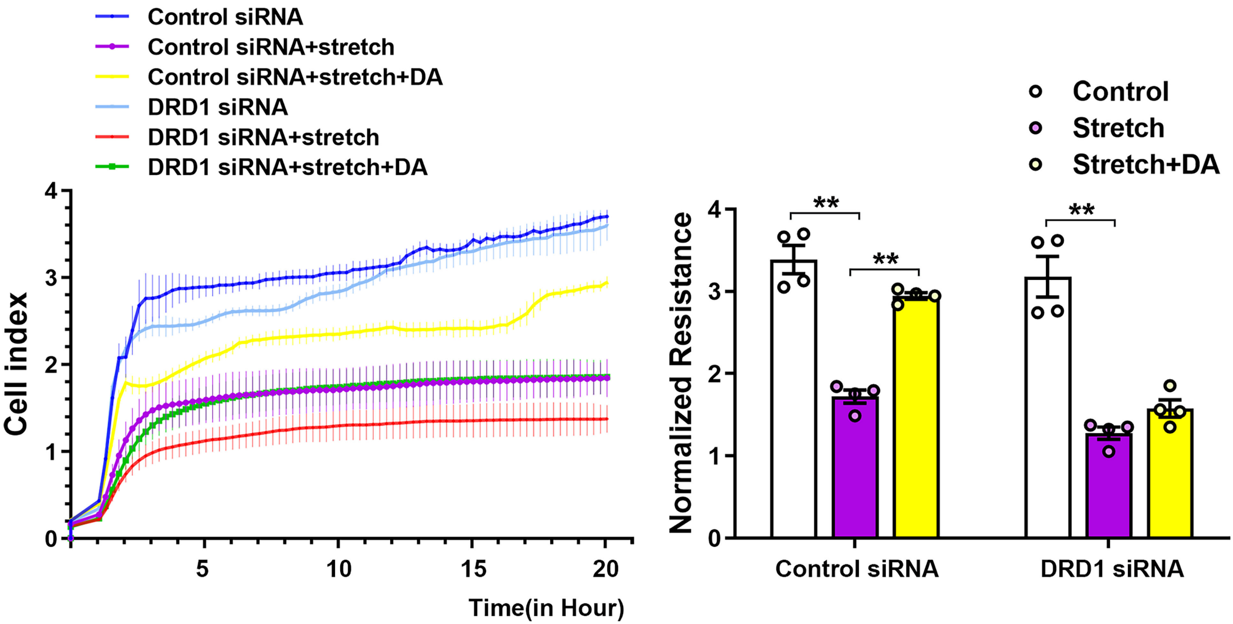
Control siRNA
Control siRNA+stretch
Control siRNA+stretch+DA
DRD1 siRNA
DRD1 siRNA+stretch
DRD1 siRNA+stretch+DA
Cell index
0
1
2
3
4
5
10
15
20
Time(in Hour)
Control
Stretch
Stretch+DA
Normalized Resistance
0
1
2
3
4
**
**
**
Control siRNA
DRD1 siRNA

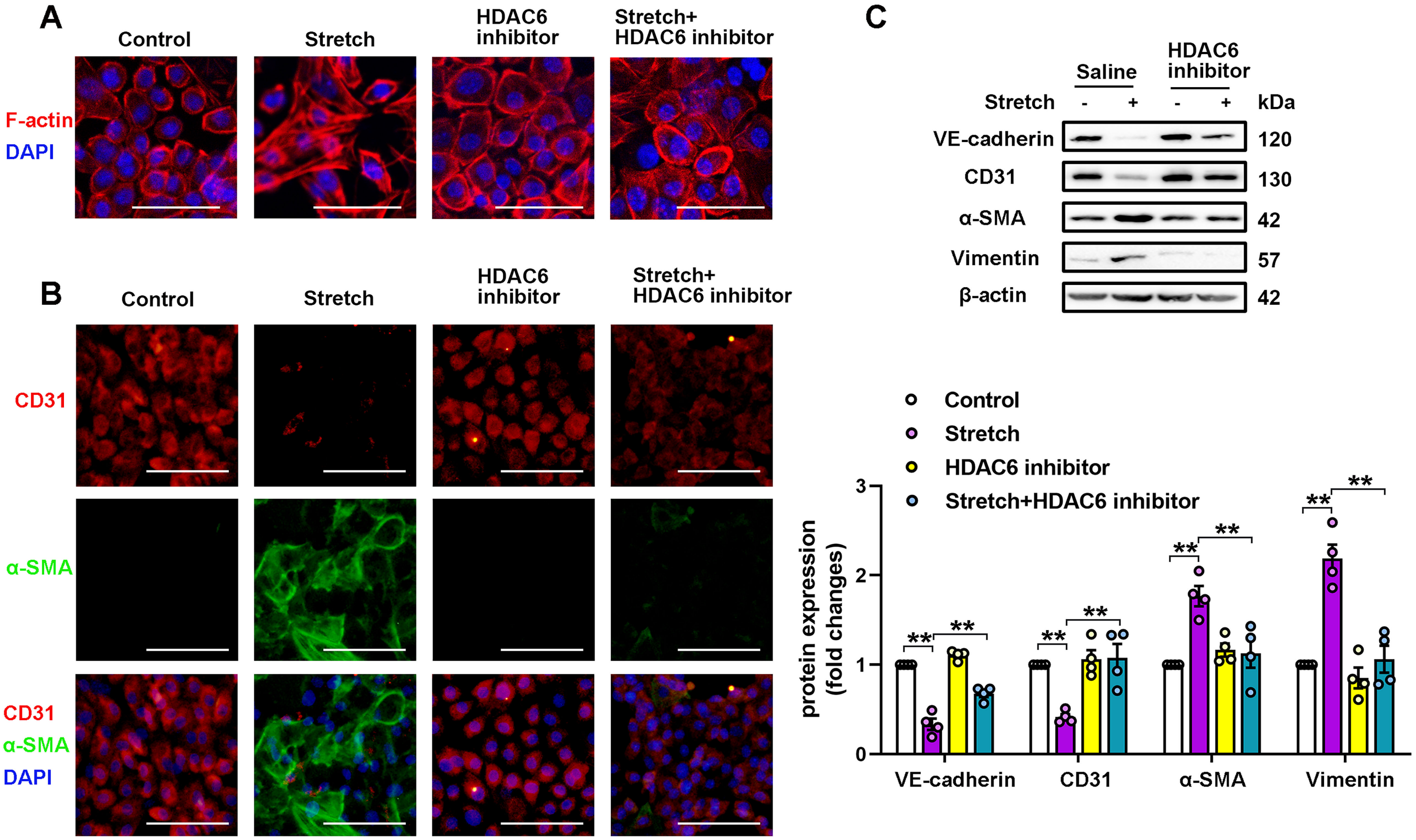
A
Control
Stretch
HDAC6 inhibitor
Stretch+ HDAC6 inhibitor
F-actin
DAPI
B
Control
Stretch
HDAC6 inhibitor
Stretch+ HDAC6 inhibitor
CD31
α-SMA
CD31
α-SMA
DAPI
C
Saline
HDAC6 inhibitor
Stretch - + - +
kDa
VE-cadherin 120
CD31 130
α-SMA 42
Vimentin 57
β-actin 42
Control
Stretch
HDAC6 inhibitor
Stretch+HDAC6 inhibitor
protein expression (fold changes)
0
1
2
3
**
VE-cadherin
CD31
α-SMA
Vimentin

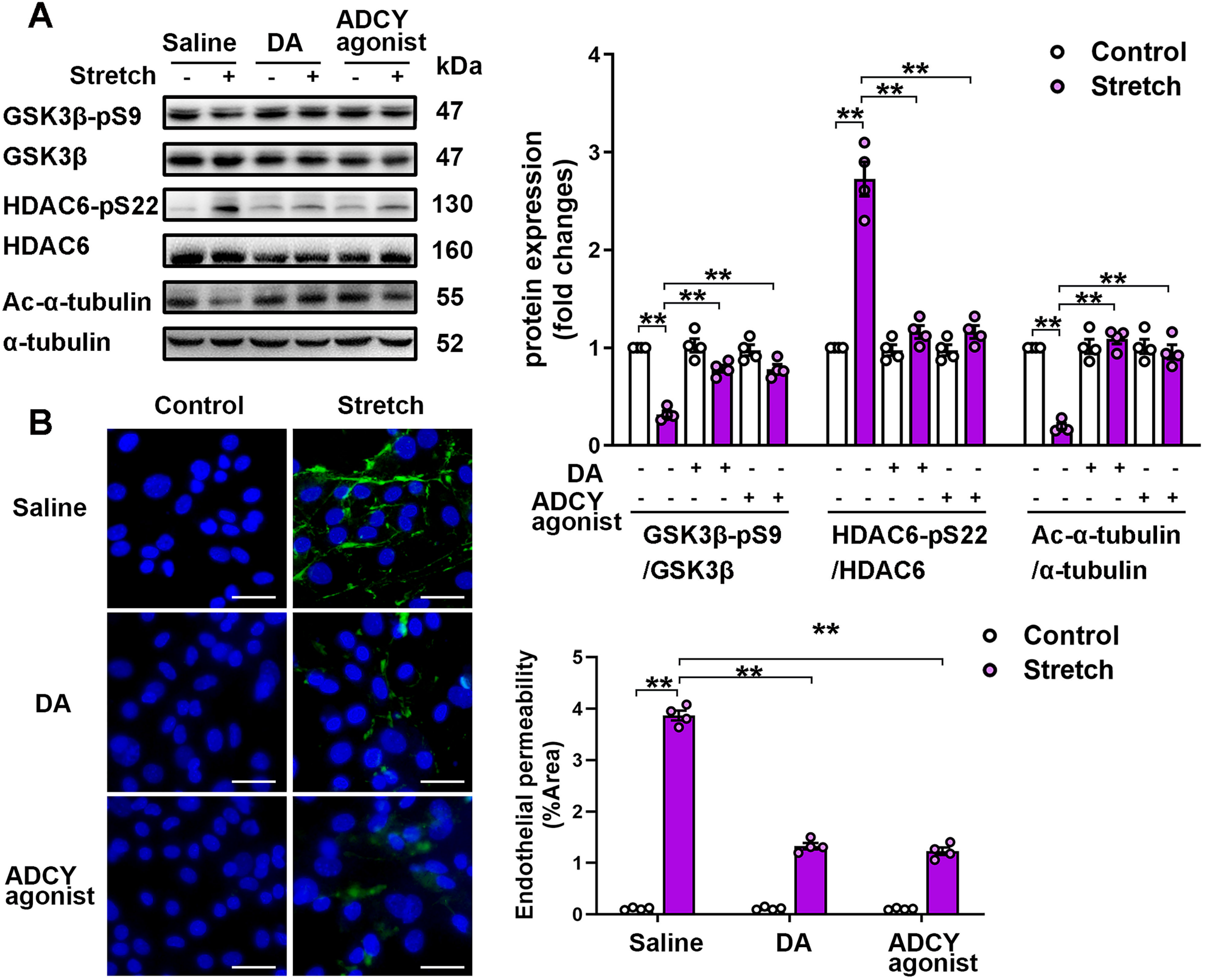
A
Saline
DA
ADCY agonist
Stretch - + - + - +
kDa
GSK3β-pS9 47
GSK3β 47
HDAC6-pS22 130
HDAC6 160
Ac-α-tubulin 55
α-tubulin 52
protein expression (fold changes)
Control
Stretch
DA
ADCY agonist
GSK3β-pS9 /GSK3β
HDAC6-pS22 /HDAC6
Ac-α-tubulin /α-tubulin
B
Control
Stretch
Saline
DA
ADCY agonist
Endothelial permeability (%Area)
Control
Stretch
Saline
DA
ADCY agonist

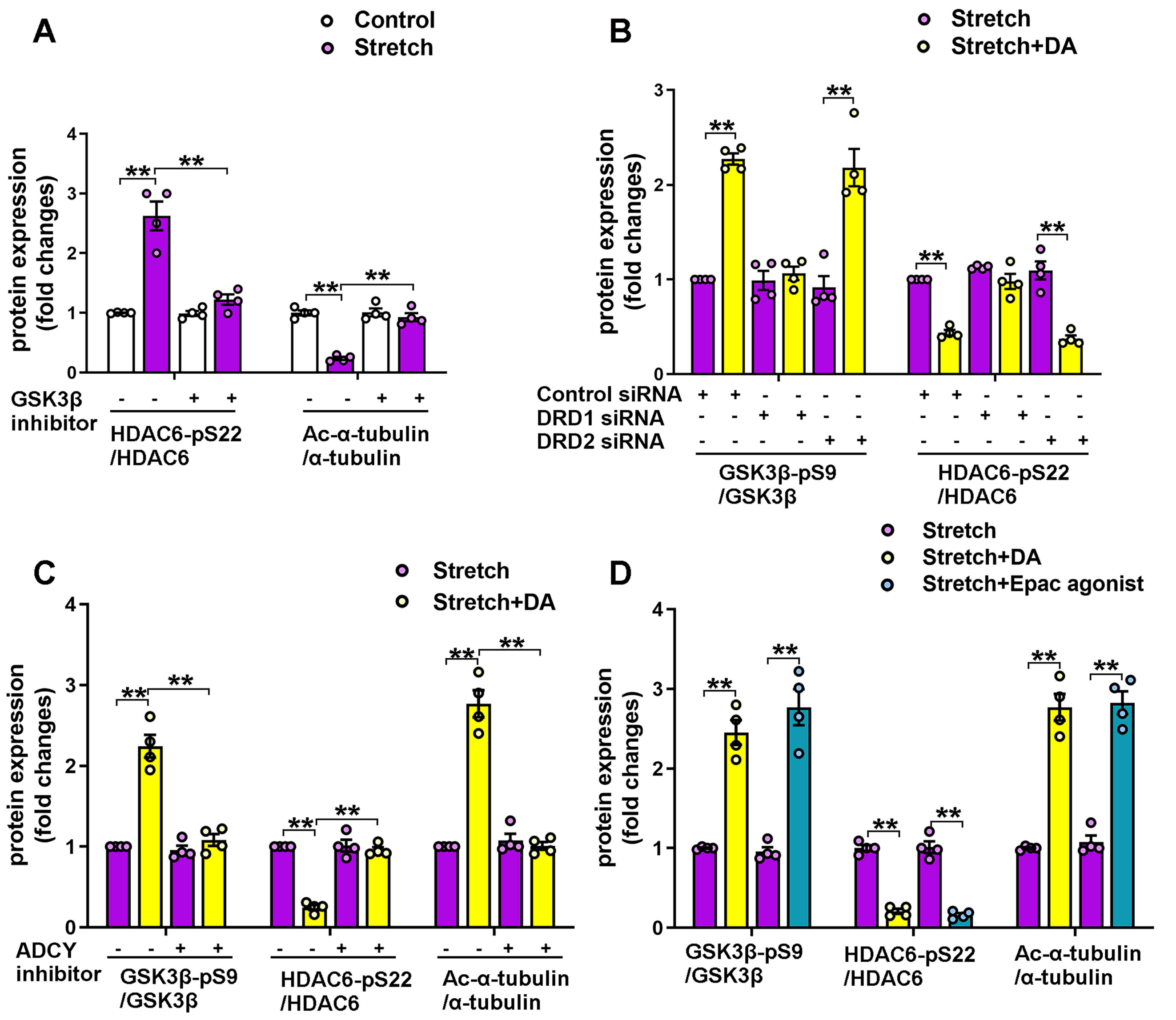
A
Control
Stretch
protein expression (fold changes)
0
1
2
3
4
**
**
**
**
GSK3β inhibitor
- - + +
- - + +
HDAC6-pS22 /HDAC6
Ac-α-tubulin /α-tubulin
B
Stretch
Stretch+DA
protein expression (fold changes)
0
1
2
3
**
**
**
**
Control siRNA
DRD1 siRNA
DRD2 siRNA
GSK3β-pS9 /GSK3β
HDAC6-pS22 /HDAC6
C
Stretch
Stretch+DA
protein expression (fold changes)
0
1
2
3
4
**
**
**
**
**
**
ADCY inhibitor
- - + +
- - + +
- - + +
GSK3β-pS9 /GSK3β
HDAC6-pS22 /HDAC6
Ac-α-tubulin /α-tubulin
D
Stretch
Stretch+DA
Stretch+Epac agonist
protein expression (fold changes)
0
1
2
3
4
**
**
**
**
**
**
GSK3β-pS9 /GSK3β
HDAC6-pS22 /HDAC6
Ac-α-tubulin /α-tubulin

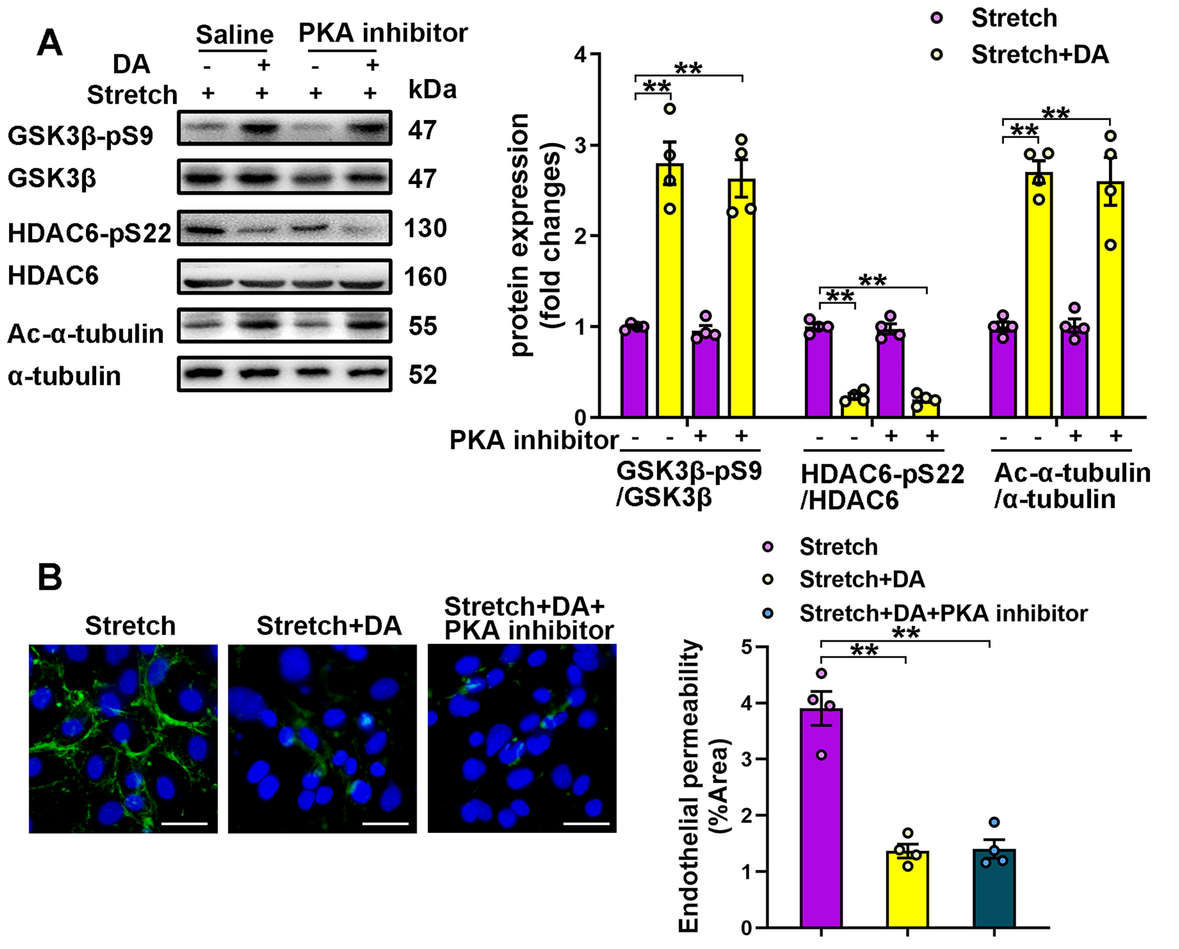

A
Saline
PKA inhibitor
DA - + - +
Stretch + + + +
kDa
GSK3β-pS9 47
GSK3β 47
HDAC6-pS22 130
HDAC6 160
Ac-α-tubulin 55
α-tubulin 52
Stretch
Stretch+DA
protein expression (fold changes)
PKA inhibitor
GSK3β-pS9 /GSK3β
HDAC6-pS22 /HDAC6
Ac-α-tubulin /α-tubulin
B
Stretch
Stretch+DA
Stretch+DA+ PKA inhibitor
Stretch
Stretch+DA
Stretch+DA+PKA inhibitor
Endothelial permeability (%Area)

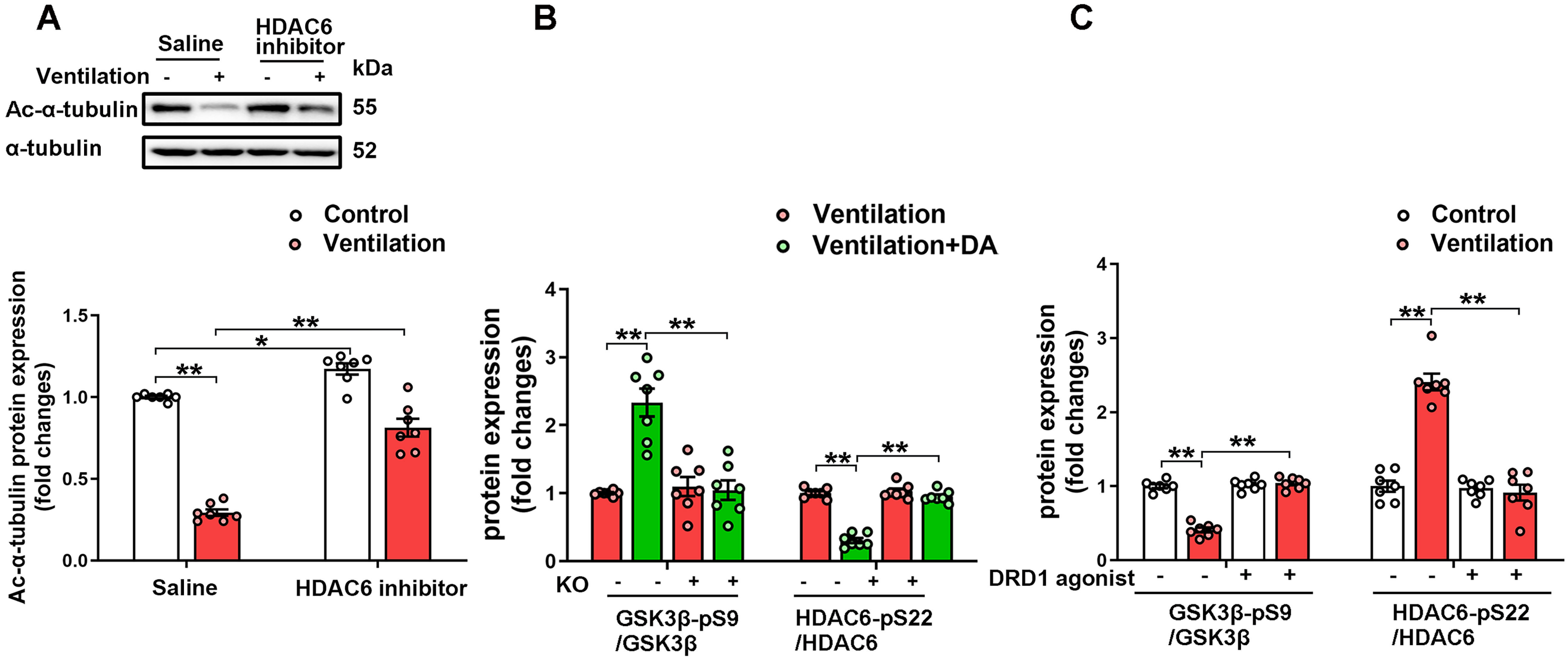

A
Saline
HDAC6 inhibitor
Ventilation - + - +
kDa
Ac-α-tubulin
55
α-tubulin
52
Control
Ventilation
Ac-α-tubulin protein expression (fold changes)
1.5
1.0
0.5
0.0
**
*
**
Saline
HDAC6 inhibitor
B
Ventilation
Ventilation+DA
protein expression (fold changes)
4
3
2
1
0
**
**
**
**
KO - - + + - - + +
GSK3β-pS9 /GSK3β
HDAC6-pS22 /HDAC6
C
Control
Ventilation
protein expression (fold changes)
4
3
2
1
0
**
**
**
**
DRD1 agonist - - + + - - + +
GSK3β-pS9 /GSK3β
HDAC6-pS22 /HDAC6

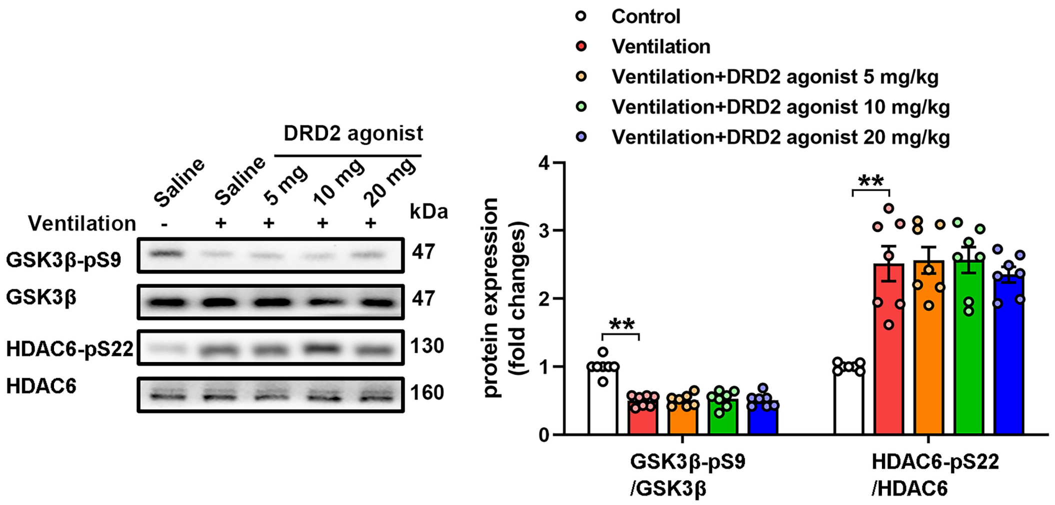
Control
Ventilation
Ventilation+DRD2 agonist 5 mg/kg
Ventilation+DRD2 agonist 10 mg/kg
Ventilation+DRD2 agonist 20 mg/kg
DRD2 agonist
Saline
Saline
5 mg
10 mg
20 mg
kDa
Ventilation - + + + +
GSK3β-pS9 47
GSK3β 47
HDAC6-pS22 130
HDAC6 160
protein expression (fold changes)
**
**
GSK3β-pS9 /GSK3β
HDAC6-pS22 /HDAC6

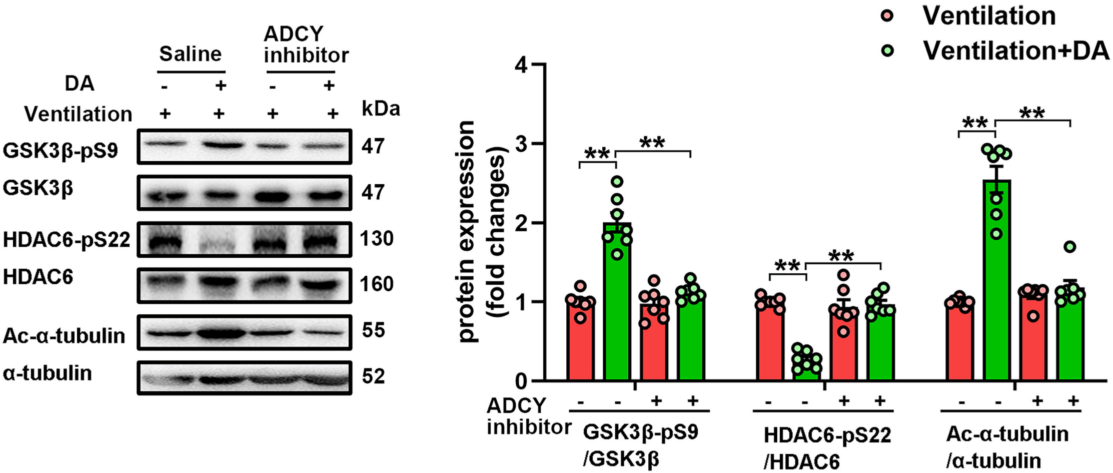
Saline
ADCY inhibitor
DA
Ventilation
kDa
GSK3β-pS9
GSK3β
HDAC6-pS22
HDAC6
Ac-α-tubulin
α-tubulin
47
47
130
160
55
52
Ventilation
Ventilation+DA
protein expression (fold changes)
ADCY inhibitor
GSK3β-pS9 /GSK3β
HDAC6-pS22 /HDAC6
Ac-α-tubulin /α-tubulin

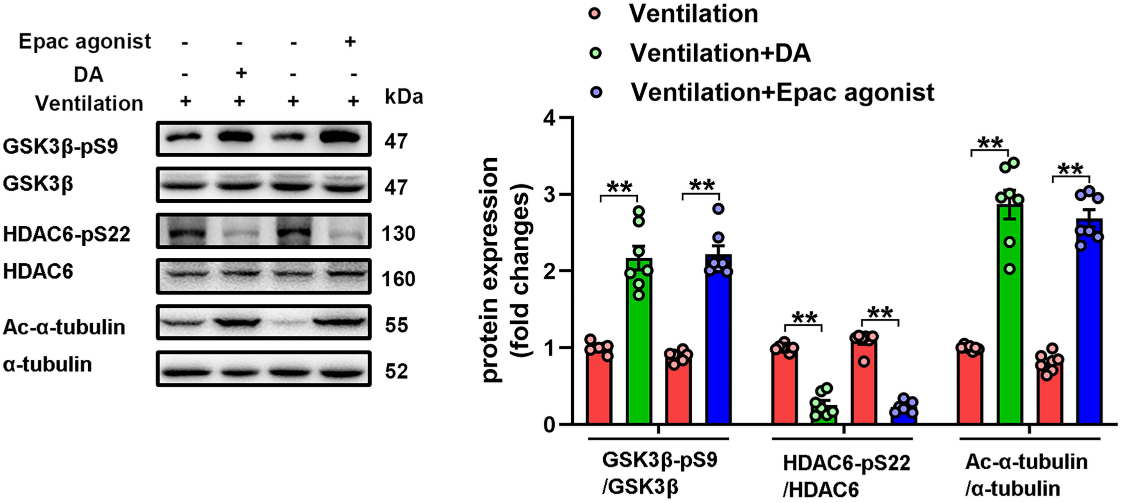

Epac agonist - - - +
DA - + - -
Ventilation + + + +
kDa
GSK3β-pS9 47
GSK3β 47
HDAC6-pS22 130
HDAC6 160
Ac-α-tubulin 55
α-tubulin 52
Ventilation
Ventilation+DA
Ventilation+Epac agonist
protein expression (fold changes)
0
1
2
3
4
**
GSK3β-pS9 /GSK3β
HDAC6-pS22 /HDAC6
Ac-α-tubulin /α-tubulin

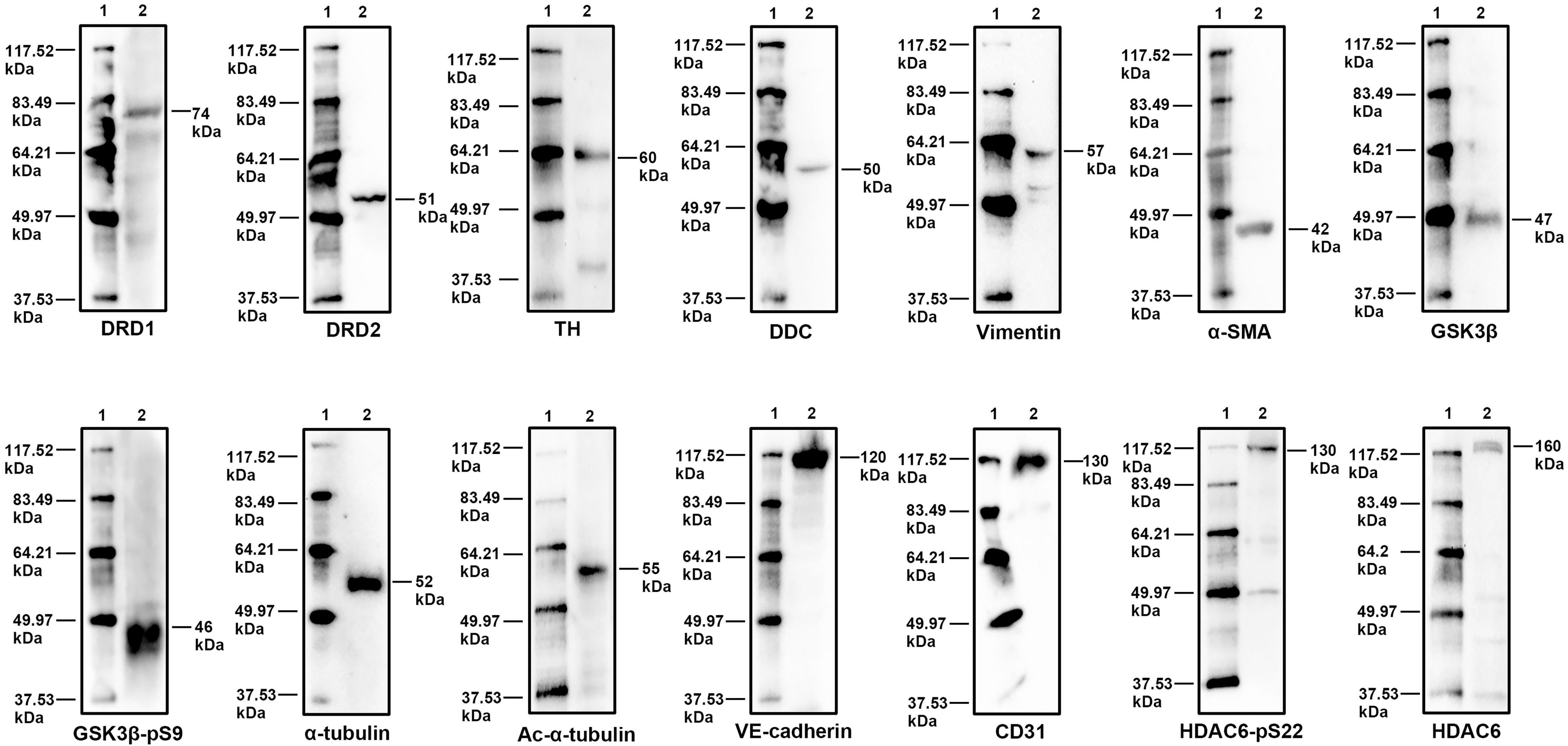
1 2
117.52 kDa
83.49 kDa
64.21 kDa
49.97 kDa
37.53 kDa
74 kDa
DRD1
51 kDa
DRD2
60 kDa
TH
50 kDa
DDC
57 kDa
Vimentin
42 kDa
α-SMA
47 kDa
GSK3β
46 kDa
GSK3β-pS9
52 kDa
α-tubulin
55 kDa
Ac-α-tubulin
120 kDa
VE-cadherin
130 kDa
CD31
130 kDa
HDAC6-pS22
64.2 kDa
160 kDa
HDAC6
